# Spatially Informed Graph Structure Learning Extracts Insights from Spatial Transcriptomics

**DOI:** 10.1002/advs.202403572

**Published:** 2024-10-09

**Authors:** Wan Nie, Yingying Yu, Xueying Wang, Ruohan Wang, Shuai Cheng Li

**Affiliations:** ^1^ Department of Computer Science City University of Hong Kong Hong Kong SAR China; ^2^ City University of Hong Kong (Dongguan) Dongguan 523000 China

**Keywords:** graph structure learning, cell‐cell interactions, spatial clustering, spatial transcriptomics

## Abstract

Embeddings derived from cell graphs hold significant potential for exploring spatial transcriptomics (ST) datasets. Nevertheless, existing methodologies rely on a graph structure defined by spatial proximity, which inadequately represents the diversity inherent in cell‐cell interactions (CCIs). This study introduces STAGUE, an innovative framework that concurrently learns a cell graph structure and a low‐dimensional embedding from ST data. STAGUE employs graph structure learning to parameterize and refine a cell graph adjacency matrix, enabling the generation of learnable graph views for effective contrastive learning. The derived embeddings and cell graph improve spatial clustering accuracy and facilitate the discovery of novel CCIs. Experimental benchmarks across 86 real and simulated ST datasets show that STAGUE outperforms 15 comparison methods in clustering performance. Additionally, STAGUE delineates the heterogeneity in human breast cancer tissues, revealing the activation of epithelial‐to‐mesenchymal transition and PI3K/AKT signaling in specific sub‐regions. Furthermore, STAGUE identifies CCIs with greater alignment to established biological knowledge than those ascertained by existing graph autoencoder‐based methods. STAGUE also reveals the regulatory genes that participate in these CCIs, including those enriched in neuropeptide signaling and receptor tyrosine kinase signaling pathways, thereby providing insights into the underlying biological processes.

## Graphical abstract

STAGUE is an innovative framework that introduces graph structure learning into unsupervised representation learning of spatial transcriptomics data. It generates a learned adjacency matrix and spatially aware cell embeddings for the cell graph. The adjacency matrix can discern novel cell‐cell interactions, whereas the cell embeddings support multiple downstream applications such as clustering, visualization, and trajectory inference.

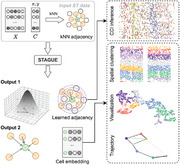

## 1 Introduction

Within the tissues of multicellular organisms, cells often aggregate into spatially organized and functionally distinct anatomical structures,^[^ ^1^, ^2^ ^]^ and proximal cells frequently coordinate their biological functions through cell‐cell interactions (CCIs). The dual challenges of pinpointing these spatial clusters, i.e., spatial clustering, and decoding the intricate communication patterns between cells, i.e., CCI inference, are pivotal for a comprehensive understanding of biological mechanisms. The groundbreaking spatial transcriptomic (ST) technologies,^[^ ^3^ ^]^ which allow for gene expression profiling while retaining critical spatial localization information, present an invaluable opportunity to discover spatially continuous clusters and decipher CCIs with greater biological fidelity. Recently, there has been great interest in applying unsupervised graph representation learning (UGRL) techniques to integrate spatial information into the learning of cell embeddings and spatial clustering.^[^ ^4^, ^5^, ^6^, ^7^, ^8^, ^9^, ^10^, ^11^ ^]^ These methods involve converting the input ST data into a cell graph, where cells or spots are treated as nodes. Edges between these nodes are established based on the spatial proximity of the corresponding cells. By applying UGRL algorithms, they seek to infer discriminative, low‐dimensional representations of cells, which are intended to preserve the essential spatial and gene expression information in the ST data. The inferred low‐dimensional cell representations provide a powerful basis for downstream clustering with unsupervised algorithms such as k‐means,^[^ ^12^ ^]^ mclust,^[^ ^13^ ^]^ and Leiden.^[^ ^14^ ^]^ Among these methods, some propose jointly optimizing spatially aware cell representations while inferring novel CCIs.^[^ ^9^, ^10^, ^11^ ^]^ They build upon graph autoencoders (GAE), learning cell representations by reconstructing the predefined adjacency matrix of the input cell graph. Subsequently, novel CCIs are identified by analyzing the newly generated edges according to the reconstructed adjacency matrix.

Recently, graph contrastive learning (GCL) has emerged as an important UGRL paradigm for ST studies.^[^ ^15^ ^]^ Existing GCL‐based methods^[^ ^16^, ^17^, ^18^, ^19^, ^20^ ^]^ build upon the Deep Graph Infomax (DGI) framework.^[^ ^21^ ^]^ They typically utilize ad‐hoc augmentation functions^[^ ^15^ ^]^ to generate different views of the input cell graph stochastically, such as randomly shuffling node features or randomly adding/dropping edges of the original cell graph. Subsequently, they harness the principle of mutual information maximization to bring similar views closer while distancing the irrelevant ones. It is crucial to note that in current GCL‐based methods, the generation of different cell graph views relies on a predefined graph structure based on spatial proximity. When constructing the graph topology, these methods often identify neighboring cells based on two main criteria: proximity within a designated distance or a predetermined quantity of the nearest cells. This methodology may possess certain intrinsic limitations, as merely factoring in spatial proximity does not adequately capture the complexity of intercellular communications that regulate gene expression dependencies among cells. For instance, the signal strength from cell secreting signaling molecules (e.g., protein ligands) often attenuates with increasing distance,^[^ ^22^ ^]^ and nearby cells selectively respond to these molecules via specific receptors.^[^ ^23^ ^]^ A smaller distance range for constructing cell graphs can reduce the inclusion of irrelevant interactions, known as noise, but it may also overlook significant long‐distance relationships. Conversely, a larger range might capture these interactions at the cost of reducing the signal‐to‐noise ratio. Therefore, depending solely on spatial information to define the graph structure is insufficient and challenging. Moreover, this trade‐off poses a limitation on existing GAE‐based methods for CCI inference, as their reconstructing objective, i.e., the predefined cell graph adjacency matrix, may inherently contain noise.

To address the above problems, in this study, we investigate a scenario where the cell graph structure is dynamically learned and adjusted during the contrastive learning process. To this end, we draw inspiration from recent developments in graph structure learning (GSL),^[^ ^24^, ^25^, ^26^ ^]^ and propose STAGUE, an unsupervised representation learning model for **S**patial **T**ranscriptomics with sp**A**tially informed **G**raph str**U**cture l**E**arning. STAGUE infers discriminative cell representations by exploiting the contrast between multiple views of the input cell graph. By parameterizing and optimizing the cell graph structure, it can generate learnable graph views to enhance the efficiency of the contrast process. This approach also seamlessly integrates the spatial clustering and CCI inference tasks into a unified framework. Specifically, STAGUE employs a spatial learner module that effectively models a cell graph adjacency matrix by utilizing the intrinsic spatial and gene expression relationships among cells. The learned adjacency matrix captures the statistical dependencies among cells, reflecting their proximities and potential interactions. The integration of the normalized temperature‐scaled cross‐entropy loss^[^ ^25^, ^27^ ^]^ and triplet loss as joint contrastive objectives facilitates the contrast between different views. Benchmark results show that STAGUE outperforms 15 comparison methods in spatial clustering across 86 ST datasets from different platforms. Moreover, STAGUE exhibits the capability to discern heterogeneity within both cancerous and paracancerous regions in human breast cancer samples. It uncovers the localized initiation of the epithelial‐to‐mesenchymal transition as well as the activation of PI3K/AKT signaling pathways in particular sub‐regions. For the CCI inference task, STAGUE shows superior ability to discern biologically meaningful CCIs compared to existing GAE‐based methods, and the regulatory genes governing these interactions are revealed. Comprehensive parameter analyses and ablation studies validate the effectiveness of the proposed components in STAGUE.

## 2 Results

### 2.1 Overview of STAGUE

**Figure 1:**
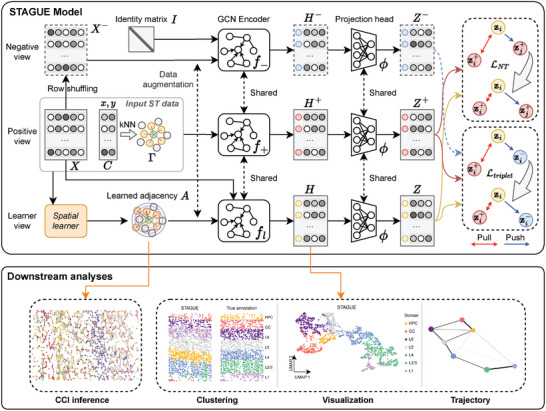
Overview of STAGUE. The input ST data comprises gene expression matrix *X* and spatial coordinates *C*. Utilizing *C*, a raw kNN cell adjacency matrix Γ is derived. STAGUE processes the input ST data through three views: the learner view, which combines expression features with spatial information to infer a cell adjacency matrix *A* capturing cell dependencies; the positive view, derived from the raw kNN cell adjacency matrix Γ; and the negative view, formed by shuffling cell features and using an identity adjacency matrix. The three views undergo data augmentation and are subsequently encoded using GCN encoders with shared weights. The embeddings are then projected to compute contrastive loss, utilizing a joint objective composed of the NT‐Xent loss $L_{NT}$ and triplet loss $L_{triplet}$. The low‐dimensional embedding of the learner view *H* can be used for multiple downstream tasks, including clustering, visualization, and trajectory analysis, whereas the learned adjacency matrix *A* aids in identifying novel CCIs.

STAGUE is an unsupervised representation learning model for spatial transcriptomics (ST), utilizing spatially informed graph structure learning (GSL) to integrate spatial clustering and cell‐cell interaction (CCI) inference tasks into a unified framework (**Figure** **1**, Experimental Section). Given an ST dataset as input, we can extract a gene expression matrix and spatial coordinates of cells. Utilizing the coordinates, we derive a raw k‐nearest neighbors (kNN) cell adjacency matrix. STAGUE mainly consists of three different views of the input ST data. The learner view is derived using a spatial learner module that integrates the expression features and spatial information to learn a cell adjacency matrix. Compared with the raw kNN cell adjacency matrix, the learned adjacency matrix features continuous values and is designed to capture the statistical dependencies among cells, reflecting their proximities and potential interactions. The positive view is generated from the raw kNN cell adjacency matrix and serves as the positive pair of the learner view. The negative view is formed by randomly shuffling the node features and using an identity matrix as the adjacency matrix. Before the Graph Convolutional Network (GCN)^[^ ^28^ ^]^ encoding, the three views are processed using data augmentation techniques, including feature masking and edge dropping. The encoded embeddings are then mapped to the space where contrastive loss is applied through projection heads. The integration of the normalized temperature‐scaled cross‐entropy (NT‐Xent) loss^[^ ^25^, ^27^ ^]^ and triplet loss enables an effective contrast between the three views. The learned adjacency matrix from the spatial learner module can be utilized to discern novel CCIs. Furthermore, the embeddings from the learner view can be used for multiple downstream analyses, including spatial clustering, data visualization, and trajectory inference. Note that various unsupervised clustering algorithms^[^ ^12^, ^13^, ^14^ ^]^ are applicable to cell embeddings; in this study, the standard k‐means was utilized as the default clustering algorithm.

### 2.2 STAGUE Outperforms State‐of‐the‐Art Methods on Spatial Clustering

#### 2.2.1 Clustering Accuracy

In this section, we evaluate the spatial clustering accuracy of STAGUE, benchmarking it against fifteen recent algorithms using comprehensive datasets that includes both real and simulated ST datasets (Experimental Section).

The selected comparison algorithms encompass deep learning methods, both with and without the implementation of graph contrastive learning (GCL), as well as the latest statistical learning approaches to ensure a rigorous assessment. The first group of real datasets (Real#1) includes 14 datasets derived from a variety of technological platforms, including STARmap, MERFISH, osmFISH, Stereo‐seq, and 10x Visium. Among these data, eight are characterized by single‐cell resolution, while six offer spot‐level resolution. This selection presents a varied scope regarding spatial resolution, cell counts, and gene coverage. The second group of real datasets (Real#2) consists of 16 datasets selected from a recent benchmark study focused on spatial clustering.^[^ ^29^ ^]^ These data involve non‐brain tissues and cover smaller, discrete tissue domains such as breast cancer, liver, and pancreatic ductal adenocarcinoma. The first simulated dataset group (Simulated#1) is comprised of 28 datasets generated using the spatial pattern preserving simulation tool, SRTsim.^[^ ^30^ ^]^ These simulations utilized the Real#1 group as references, incorporating both tissue‐based and domain‐specific simulations. Finally, the second simulated dataset group (Simulated#2) contains 28 datasets created with the cell pseudo‐space reconstruction tool, scSpace.^[^ ^31^ ^]^ For these simulations, we explored various configurations of cell clusters and subclusters to generate the data.

To quantify the clustering performance, we employed two conventional clustering metrics: the Adjusted Rand Index (ARI)^[^ ^32^ ^]^ and Adjusted Mutual Information (AMI).^[^ ^33^ ^]^ Elevated values of these metrics denote superior performance. To mitigate the influence of randomness and ensure a fair comparison, all deep learning algorithms were executed five times, with the mean performance being reported. Further details regarding the parameters and experimental configuration for STAGUE and comparison methods are provided in the Supporting Information.

**Figure 2:**
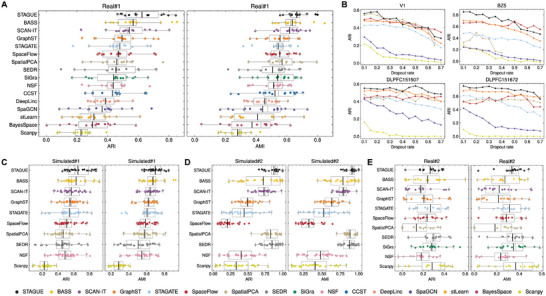
STAGUE achieves the best overall performance in spatial clustering. Comparison of clustering methods across four dataset groups using ARI (left) and AMI (right): A) Real#1, C) Simulated#1, D) Simulated#2, and E) Real#2. Each point represents the result of the corresponding method on one dataset. The black center line indicates the mean value across all datasets. Boxplot: center line, the median; upper and lower edges, the interquartile range; whiskers, 1.5 × interquartile range. B) ARI performance of selected representative methods under different dropout rates, ranging from 0.1 to 0.7 with a step of 0.05. See Figure S4 (Supporting Information) for the corresponding AMI performance. Datasets include V1 from mouse primary visual cortex, BZ5 from mouse medial prefrontal cortex, and slices #151507 and #151672 from human dorsolateral prefrontal cortex (DLPFC) (see Experimental Section). SpatialPCA failed at higher dropout rates for V1 and BZ5.

The benchmark results on the Real#1 group show that STAGUE significantly outperforms all competing methods (Wilcoxon signed‐rank test, *p*‐value < 0.05), achieving the highest mean ARI and AMI scores of 0.624 and 0.665, respectively (**Figure** **2**A). This marks an 10.13% increase in ARI and a 4.62% rise in AMI over the second‐best method, BASS.^[^ ^34^ ^]^ Scanpy performs the worst due to its disregard for spatial information. See Figures  S1 and S2 (Supporting Information) for the detailed results on each dataset. Next, the robustness of the algorithms to low sequencing depths was evaluated by systematically degrading the quality of the input ST data. We progressively introduced dropout rates from 0.1 to 0.7 in the count matrix, creating a series of downsampled datasets from four representative data in Real#1 (Figure S3, Supporting Information). Figure 2B demonstrates that STAGUE maintains a relatively superior performance across varying dropout rates for the DLPFC datasets. Nevertheless, its advantage is less pronounced in datasets with lower gene coverage, such as V1 and BZ5, particularly at higher dropout rates beyond 0.4. This could be attributed to the fact that a high dropout rate can severely reduce the signal‐to‐noise ratio, and datasets with low gene coverage are more prone to the downsampling impact.

For other benchmark dataset groups, we evaluated the top ten performing methods along with Scanpy, which served as the non‐spatial baseline. SiGra^[^ ^35^ ^]^ was evaluated only on Real#2 due to the lack of tissue images in the simulated datasets. In the Simulated#1 group (Figure 2C), STAGUE significantly outperforms all other methods (*p*‐value < 0.05). Similarly, in the Simulated#2 group (Figure 2D), STAGUE surpasses most methods by achieving statistically significant higher scores (*p*‐value < 0.05). Exceptions include SpatialPCA^[^ ^36^ ^]^ and SEDR,^[^ ^5^ ^]^ where STAGUE still posts higher mean ARI and AMI values, though these differences are not statistically significant.

Real#2 differs significantly from both Real#1 and Simulated#1/#2 by featuring smaller, noncontinuous domains without layered patterns. In this dataset group, all methods yield low ARI and AMI scores, approximately 0.3, as illustrated in Figure 2E. Among the methods that utilize spatial information, SEDR, SiGra, STAGUE, and STAGATE^[^ ^7^ ^]^ generally outperform the others. Notably, the non‐spatial method, Scanpy, not only competes well with spatial approaches in terms of ARI but also achieves the highest AMI score of all methods evaluated on Real#2. This outcome underscores the challenges of effectively utilizing spatial information to discern the subtle spatial heterogeneity in tissues with smaller, noncontinuous domains. Notably, SiGra, which leverages image data from tissues, demonstrates improved performance ranking in Real#2 compared to Real#1. This enhancement could be attributed to the consistent availability of hematoxylin and eosin (H&E) images in Real#2, in contrast to Real#1 where only spot‐level data are paired with H&E images, resulting in only modest performance for SiGra. These results suggest that the integration of tissue morphology information substantially enhances the delineation of spatial heterogeneity in tissues characterized by smaller domains.

#### 2.2.2 Scalability Analysis

Recent advancements in ST technologies have significantly increased the capacity for sequencing large numbers of cells/spots, a trend poised to continue. This underscores the importance of assessing a clustering method's effectiveness with large datasets. Here, following Yuan et al.'s benchmark study,^[^ ^29^ ^]^ we performed a scalability analysis of the leading methods using ten slices from a large‐scale MERFISH dataset,^[^ ^37^ ^]^ comprising 87 305 cells and 374 genes. Scanpy was also tested as the non‐spatial reference. This analysis examined how computational time scales with an increasing number of slices/cells under the same memory allocation (Experimental Section).

The results demonstrate that Scanpy is the most efficient, processing all ten slices within one minute (Figure S5, Supporting Information). Deep learning methods markedly surpass BASS in terms of efficiency. For example, these methods can process five slices (comprising 40 028 cells) in less than 8 min, while BASS requires 46 min. Among the deep learning approaches, GraphST is limited to processing five slices and SEDR to seven slices, both constrained by CPU memory (64 GB). STAGATE, on the other hand, is limited to five slices due to CUDA memory restrictions (24 GB) but can handle up to nine slices utilizing CPU memory. Notably, only SCAN‐IT and STAGUE can process all ten slices, with STAGUE nearly matching the performance of Scanpy by requiring less than 2 min to process the entire dataset. Overall, STAGUE demonstrates superior computational efficiency among the leading spatially informed methods, confirming its strong potential for applications on large‐scale datasets.

### 2.3 STAGUE Better Demarcates the Laminar Structure of the Mouse Primary Visual Cortex and Olfactory Bulb

This section examines the clustering efficacy of STAGUE on single‐cell resolution ST data, encompassing the investigation of differentially expressed genes, visualization of embeddings, and trajectory inference. To this end, we selected two datasets as representative examples: one from the mouse primary visual cortex (V1) acquired through STARmap, and the other from the mouse olfactory bulb obtained via Stereo‐seq.

**Figure 3:**
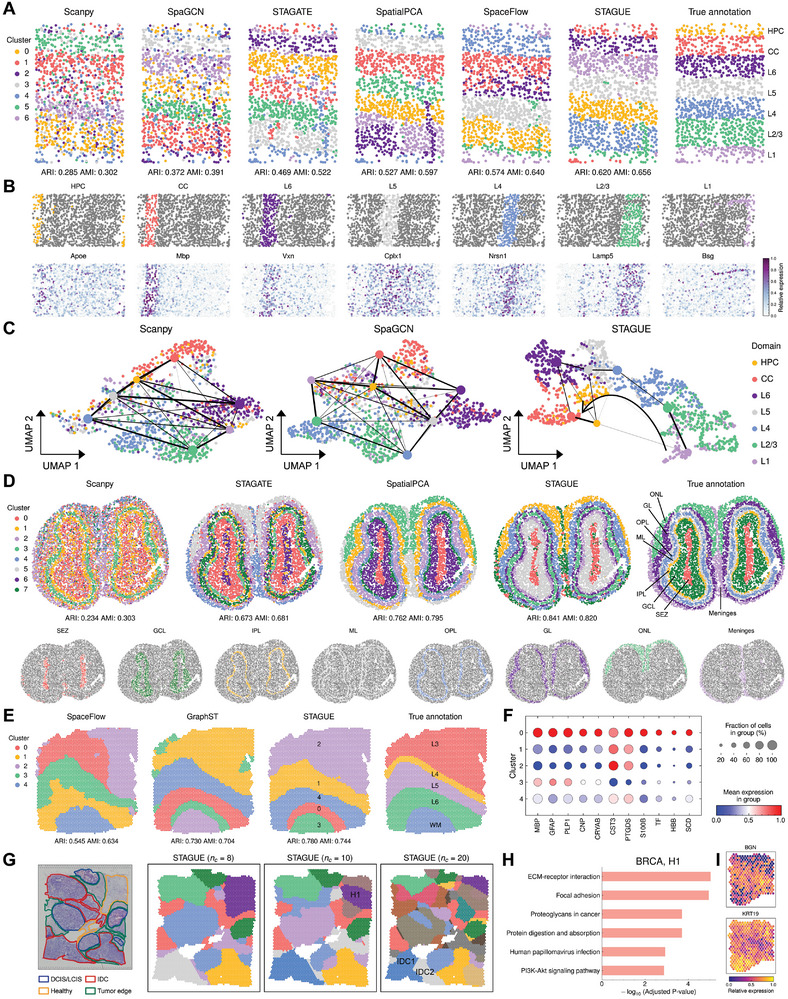
STAGUE better demarcates the laminar structure of mouse brain tissue and dissects finer‐grained structures for human brain and breast cancer tissues. A) Clustering results of different methods on the STARmap mouse V1 dataset and true annotations. The result from a single run of each method is selected for demonstration. See Figure S6 (Supporting Information) for the demonstration of other methods. B) Expression pattern of DEGs in different clusters detected by STAGUE. C) UMAP plots with ground‐truth labels overlaid with PAGA trajectory inference results using latent embeddings from Scanpy, SpaGCN, and STAGUE. D) Top panel: Clustering results of different methods on the Stereo‐seq mouse olfactory bulb dataset and true annotations. See Figure S6 (Supporting Information) for the results of other methods. Bottom panel: Visualization of the clusters identified by STAGUE, with cluster colors adjusted to match true annotations. E) Clustering results of different methods on slice #151672 of the human DLPFC dataset and true annotations. See Figure S6 (Supporting Information) for the results of other methods. F) Dotplot of the representative DEGs of cluster 0 in comparison to other clusters. G) Clustering results of STAGUE on the 10x Visium BRCA dataset with different cluster numbers *n*_*c*_. H) Pathway enrichment analysis of the H1 sub‐region. I) Expression pattern of the most significantly upregulated and downregulated genes in H1.

**Figure** **3**A illustrates that the V1 dataset exhibits a layered pattern, including hippocampus (HPC), corpus callosum (CC), and six neocortical layers from L1 to L6. STAGUE outperforms other comparison methods in delineating V1's laminar organization, with a notably higher ARI of 0.620 and AMI of 0.656, as shown in Figure 3A and Figure S6 (Supporting Information). By contrast, methods like Scanpy, SpaGCN, and STAGATE exhibit suboptimal performance, with ARIs below 0.5, indicating some distortion of the layered structure. SpatialPCA and SpaceFlow demonstrate modestly superior outcomes, with ARIs of 0.527 and 0.574, respectively; however, SpatialPCA conflates layers L1 and L2/3, and SpaceFlow inadequately separates layers L5 and L6. Utilizing the clustering results from STAGUE, we further detected 18 differentially expressed genes (DEGs) in the V1 dataset, including *Apoe*, *Mbp*, *Vxn*, *Cplx1*, *Nrsn1*, *Lamp5*, and *Bsg* that showed enriched expression patterns for regions from HPC to L1 (Figure 3B; Figure S7, Supporting Information).

To qualitatively assess the representation capabilities of the learned cell embeddings, we utilized two main approaches: 1) the application of uniform manifold approximation and projection (UMAP) for low‐dimensional visualization, and 2) the use of the partition‐based graph abstraction (PAGA) algorithm^[^ ^38^ ^]^ and Monocle3^[^ ^39^ ^]^ to conduct trajectory inference. Note that both PAGA and Monocle3 utilize low‐dimensional projections of cell gene expression as inputs. In this context, latent embeddings from spatially informed methods can provide spatially aware inputs for inferring spatial trajectories. The results with PAGA (Figure 3C) demonstrates that Scanpy and SpaGCN can hardly differentiate most domains, with the exception of layers L2/3 and L4. By contrast, STAGUE delivers significantly improved visualization of distinct domains and successfully infers a nearly linear developmental trajectory from L1 to L6. The interconnection between the CC/HPC domains and layers L5/L6 could be attributed to the mix of different cell types (Figure S8, Supporting Information). By designating L1 as the root, the pseudo‐time trajectory from Monocle3 (Figure S9, Supporting Information) reveals that Scanpy exhibits divergent trajectories, whereas SpaGCN tends to follow a more linear path but separates L2/3/4 and L5/6 into distinct branches. In contrast, only STAGUE maintains an overall linear trajectory.

For the Stereo‐seq mouse olfactory bulb dataset, we referenced the original study^[^ ^40^ ^]^ to annotate its laminar structure, including the olfactory nerve layer (ONL), glomerular layer (GL), outer plexiform layer (OPL), mitral layer (ML), internal plexiform layer (IPL), granule cell layer (GCL), subependymal zone (SEZ), and meninges (Figure 3D). We compare the clustering results of STAGUE with other methods (Figure 3D; Figure S6, Supporting Information). Among all the methods, Scanpy is the least effective in delineating the laminar structure, which is attributed to its lack of consideration of spatial information. By contrast, STAGUE, SpatialPCA, and STAGATE demonstrate improved performance and can accurately differentiate between the ONL, GL, and meninges regions. STAGUE and SpatialPCA can identify a clear boundary between GCL and SEZ, whereas STAGATE exhibits a higher degree of cluster overlap. Notably, STAGUE also effectively demarcates the OPL, ML, and IPL regions. Overall, STAGUE demonstrates superior performance, establishing distinct demarcations between the various domains of the mouse olfactory bulb.

### 2.4 STAGUE Dissects Finer‐Grained Structures for Human Dorsolateral Prefrontal Cortex and Breast Cancer Tissue

To illustrate the generalization ability of STAGUE on spot‐level data, we give a detailed analysis of the clustering results on human dorsolateral prefrontal cortex (DLPFC) and human breast cancer (BRCA) tissues. Specifically, we use slice #151672 of the DLPFC dataset^[^ ^2^ ^]^ and the BRCA Block A Section 1 sample from the 10x Genomics database as representative examples.

Figure 3E illustrates that the #151672 slice contains four layers of the human dorsolateral prefrontal cortex (L3‐L6) and white matter (WM). The clustering performance of STAGUE and GraphST is generally superior to other algorithms as illustrated in both Figure 3E and Figure S6 (Supporting Information). For instance, SpaceFlow has difficulty establishing regular boundaries and fails to identify the L3 layer accurately. In contrast to SpaceFlow, STAGUE and GraphST demonstrate improved identification of L3, with STAGUE in particular providing a more precise delineation of its boundary. Notably, both STAGUE and GraphST partition L6 and WM into three discrete subdomains. Figure 3F and Figure S10 (Supporting Information) illustrates that, when compared to clusters 3 and 4, the interior cluster 0 shows elevated expression of genes such as *MBP*, *GFAP*, *PLP1*, *CNP*, etc. These genes are typically associated with myelin and glial cells, suggesting a distinct cellular composition in cluster 0. Additionally, clusters 0, 1, and 2 demonstrate elevated expression of *CST3* and *PTGDS* relative to clusters 3 and 4, suggesting a potential mixture of cell types within these clusters.

Human breast cancer exhibits high intratumoral and intertumoral heterogeneity.^[^ ^41^ ^]^ To facilitate interpreting the clustering results of the BRCA dataset, we referred to Fu et al.'s manual annotations.^[^ ^5^ ^]^ The annotation includes 20 domains, which could be grouped into four main morphotypes: ductal carcinoma in situ/lobular carcinoma in situ (DCIS/LCIS), invasive ductal carcinoma (IDC), healthy region, and tumor edge (Figure 3G). We changed the number of clusters *n*_*c*_ to evaluate STAGUE's performance in capturing the heterogeneity within cancerous and paracancerous tissues. As shown in Figure 3G, the clustering results visually agree with the manual annotations at the macroscopic level and are getting finer as *n*_*c*_ increases. When *n*_*c*_ = 10, we found that H1 is clustered as an isolated sub‐region within the healthy area located at the upper right corner. Moreover, when *n*_*c*_ = 20, the IDC region at the bottom left corner is clustered into two sub‐regions, namely IDC1 and IDC2.

Next, we performed differential expression analysis followed by pathway enrichment analysis on those identified sub‐regions (Experimental Section). The results show that the ribosome pathway is significantly upregulated in IDC1 compared to IDC2 with an adjusted *p*‐value = 3.37 × 10^−30^ (Figure S11, Supporting Information). Recent studies have shown that ribosome biogenesis is enhanced in the epithelial‐to‐mesenchymal transition (EMT), which contributes to the metastatic outgrowth of breast cancer cells.^[^ ^42^ ^]^ This indicates that the IDC1 sub‐region may exhibit increased invasive characteristics compared to IDC2. Figure 3H reveals that the sub‐region H1, compared to the other healthy sub‐regions, displays elevated activity in extracellular matrix (ECM)‐receptor interaction, focal adhesion, and phosphatidylinositol 3‐kinase (PI3K)‐Akt signaling pathways, primarily regulated by *COL1A1*, *COL1A2*, *COL6A2*, *COL6A1*, and *THBS1*. These pathways are known to be aberrantly activated in breast cancers.^[^ ^43^, ^44^, ^45^ ^]^ Meanwhile, the genes exhibiting the greatest differential expression in H1 are *BGN*, which is the most upregulated, and *KRT19*, the most downregulated (Figure 3I). Recent studies have shown marked overexpression of *BGN* in breast tumors versus normal tissues^[^ ^46^ ^]^ and significant downregulation of *KRT19* in ductal breast cancer relative to their normal counterparts.^[^ ^47^ ^]^ These results suggest the possibility that the H1 sub‐region could have undergone malignant transformations due to the adjacent tumor proliferation.^[^ ^48^ ^]^ Overall, STAGUE could detect the heterogeneity within the cancerous and paracancerous regions.

### 2.5 STAGUE Refines CCIs by Incorporating both Spatial and Gene Expression Information

In this section, we explore the characteristics of the adjacency matrix (Adj.) derived from the STAGUE model. Initially, we analyze the varying trend of Adj. values as distances increase. Subsequently, we assess how integrating gene expression data can dynamically counteract the impact of irrational spatial constraints, thereby enhancing the validity of the learned Adj.

#### 2.5.1 CCI Strengths Attenuate with Increasing Distance

STAGUE refines the adjacency matrix of the initial spatial graph through the weighted sum of two components, as detailed in Equation (5). The first component is derived from the cosine similarity between the features of cells *i* and *j*, encoded from their gene expression profiles. This term assesses the likelihood of communication inferred from gene expression data. The second component is an exponential spatial decay term dependent on ${\hat{d}}_{ij}$, the normalized distance between cells *i* and *j*. This normalization involves dividing the actual distance, *d*_*ij*_, by a dataset‐adaptive cutoff distance, *d*_*c*_, which is determined as the median of the distances between each cell and its *k*‐th nearest neighbor. Similar methods of edge weight decay have been utilized in SpatialDE^[^ ^49^ ^]^ and SpaGCN.^[^ ^4^ ^]^ For clarity, in this study, we denote the first cosine similarity term as cos (·) and the second spatial decay term as exp (·).

**Figure 4:**
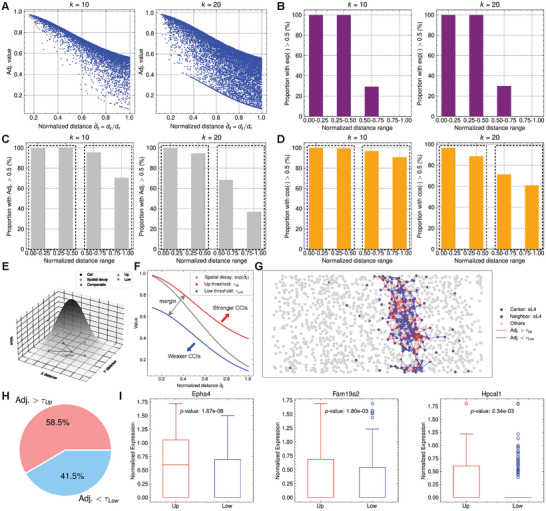
STAGUE refines the learned adjacency matrix by integrating both spatial information and gene expression data. A) Values of the learned adjacency matrix (before normalization) in the V1 dataset. Adj.: adjacency matrix. Proportion of the B) spatial decay term exp (·), C) Adj. values, and D) cosine similarity term cos (·) exceeding 0.5 across different ranges of normalized distance. E) Illustration demonstrating that the actual CCI strength may deviate from the values specified by a static spatial decay. Exponential spatial decay is chosen for this illustration. The width of the edges between cells is proportional to the CCI strength. F) Illustration of the criteria for determining stronger and weaker CCIs. Here, *k* = 20 and γ = 2. G) Stronger and weaker CCIs between eL4 cells and H) their respective ratios. I) Top upregulated genes in neighboring eL4 cells with stronger CCIs, identified using the Wilcoxon rank‐sum test.

**Figure** **4**A shows that the Adj. values decrease as the normalized distance ${\hat{d}}_{ij}$ increases, consistent with the expected influence of the spatial decay term. For different *k* values, cells within the same normalized distance range share an identical spatial decay term, even though this normalized distance might correspond to varying actual distances given different *d*_*c*_ values. For instance, consider a fixed normalized distance ${\hat{d}}_{ij}=d_{ij}/d_{c}$. A smaller *k* leads to a shorter *d*_*c*_ and thereby a shorter actual distance *d*_*ij*_, while a larger *k* indicates a longer actual distance. However, both scenarios maintain the same spatial decay due to the consistent ${\hat{d}}_{ij}$.

If we only use the spatial decay term to represent Adj., the proportion of Adj. values greater than 0.5 relative to ${\hat{d}}_{ij}$ will keep stable for different *k* values (Figure 4B). However, we find an intriguing pattern for the actual Adj. values (Figure 4C): as *k* increases, the proportion of Adj. > 0.5 remains relatively stable when ${\hat{d}}_{ij}<\text{0.5}$, contrasting sharply with the significant decrease in this proportion for ${\hat{d}}_{ij}>\text{0.5}$. This contrast drives us to inspect the cosine similarity term of Adj. values (Figure 4D). For larger *k*, we find that the ratio of cos (·) > 0.5 significantly decreases when ${\hat{d}}_{ij}>0.5$. This suggests that for small values of *k*, a normalized distance ${\hat{d}}_{ij}>0.5$ might correspond to a relatively short actual distance *d*_*ij*_. Consequently, the ligand concentration remains above a critical threshold, facilitating communication between cells (cos (·) > 0.5). Conversely, as *k* increases, the same normalized distance represents a larger actual distance. This leads to a significant decrease in ligand concentration, reducing the likelihood of cell communication (cos (·) < 0.5).

In conclusion, the model's learned cell embeddings effectively capture the actual distances between cells, causing those that are far apart to exhibit lower cosine similarity. As a result, the overall Adj. contains a smaller proportion of distant CCIs as *k* increases. This insight underscores the model's capability to dynamically reflect changes in cellular proximity and interaction likelihood based on the parameter *k*.

#### 2.5.2 Gene Expression Dynamically Rectifies Irrational Spatial Constraints

The discussion above reveals that the refined Adj. incorporates constraints based on the distances between cells. However, relying solely on this method to validate the Adj. is inadequate because it is possible to explicitly integrate spatial decay into the Adj. when defining the raw spatial graph. The selection of spatial decay functions covers a variety of methods, typically involving inverse‐squared distance decay and exponential decay with the squared distance. A shared characteristic of these models is that they are static and can impose unreasonable spatial constraints. As shown in Figure 4E, the actual CCI strength may surpass the value specified by spatial decay due to the specificity of intercellular communications,^[^ ^50^ ^]^ and vice versa. Note that the refined Adj. from STAGUE also incorporates constraints based on gene expression information. To further validate the effectiveness of the refined Adj., we explored its ability to mitigate the unreasonable spatial constraints by utilizing gene expression information.

To this end, we initially identified the CCIs that exhibit strengths either stronger or weaker than those predicted by spatial decay. To minimize the effects of randomness, a safe margin was employed to exclude adjacency values that closely align with the expected spatial decay patterns (Figure 4F). Subsequently, we analyzed the signature genes responsible for these deviations in CCI strength. See Experimental Section for details about these procedures. Figure 4G demonstrates both the stronger and weaker communications between eL4 cells. Among these, 58.5% of the CCIs are stronger, while 41.5% are weaker. Next, we categorized the neighboring eL4 cells into two groups based on their association with either upper or lower CCIs and explored the differentially expressed genes (DEGs) between these groups. Figure 4I shows three top upregulated genes in eL4 cells with stronger CCIs, including *Epha4* (ephrin receptors involved in developmental nervous system events), *Fam19a2* (chemokine‐like small secreted proteins functioning as brain‐specific chemokines or neurokines), and *Hpcal1* (calcium‐binding proteins crucial for neuronal signaling). Figure S12 (Supporting Information) illustrates spatial decay‐violating CCIs between endothelial cells and eL2/3 cells, with a predominance of weaker interactions (70.6%) compared to stronger ones (29.4%). The top upregulated genes in eL2/3 cells associated with stronger CCIs include *Nov* (involved in extracellular matrix signaling), *Hax1* (mediates neuronal differentiation by binding to copine proteins), and *B2m* (a MHC class I molecule).

In conclusion, these findings confirm that the refined Adj. from STAGUE successfully integrates gene expression information, potentially mitigating the impact of unreasonable spatial constraints.

### 2.6 STAGUE Identifies Cell‐Cell Interactions Reflecting the Established Biological Priors

Existing deep learning methods that simultaneously learn cell embeddings and infer CCIs, such as DeepLinc,^[^ ^9^ ^]^ CLARIFY,^[^ ^10^ ^]^ and TENET,^[^ ^11^ ^]^ initiate their processes by creating a raw spatial graph. Subsequently, they utilize graph autoencoder (GAE) or variational graph autoencoder (VGAE),^[^ ^51^ ^]^ frameworks to reconstruct the adjacency matrix of the raw graph. The efficacy of these models in CCI inference is assessed by the degree of resemblance between the original cell graph adjacency matrix and the reconstructed one. In a departure from this methodology, STAGUE aims to produce an adjacency matrix that is de‐noised to facilitate contrastive learning. This method anticipates a learned matrix that deviates from the original, rendering evaluation based on similarity to the initial matrix as inappropriate.

**Figure 5:**
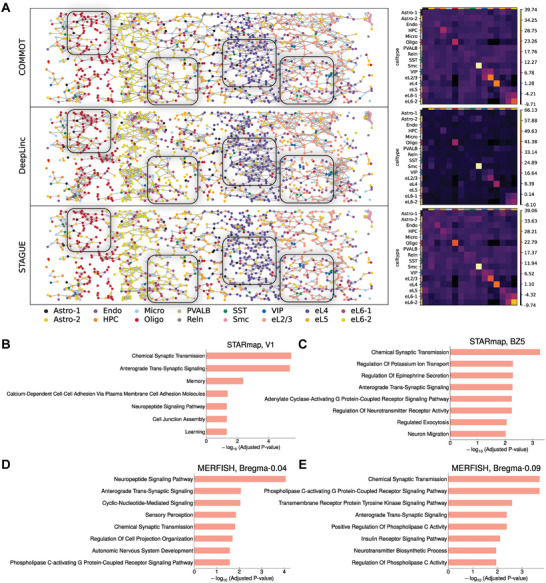
STAGUE identifies more biologically meaningful cell‐cell interactions and their regulatory genes. A) Left panel: CCI inference results on the STARmap V1 dataset. CCI visualization with edge counts–COMMOT: 5382, DeepLinc: 5380, STAGUE: 5372. The black box highlights the regions where DeepLinc's results show significant difference compared to STAGUE and COMMOT. Right panel: Neighborhood enrichment analysis between cell types, where positive scores indicate enriched CCIs. Astro, astrocytes; Endo, endothelial cells; HPC, hippocampal excitatory subtype; Micro, microglia; Oligo, oligodendrocytes; PVALB, Reln, SST, VIP, inhibitory neuron subtypes; Smc, smooth muscle cells; eL2/3, eL4, eL5, eL6, excitatory neuron subtypes. B–E) Gene Ontology biological processes enrichment analysis for the signature genes of V1, BZ5, Bregma–0.04, and Bregma–0.09 datasets.

In the context of CCI inference, assessing the accuracy of the inferred results can be challenging due to the often unknown biological ground truth.^[^ ^52^ ^]^ Here, we turn to COMMOT,^[^ ^53^ ^]^ a tool that infers CCIs using prior knowledge about signaling ligands and receptors. Kindly note that we use its results as a “reference” to assess the degree to which the adjacency matrices learned by deep learning models can reflect the established prior biological knowledge (Experimental Section). Specifically, the comparison between DeepLinc, CLARIFY, TENET and STAGUE on the mouse primary visual cortex (V1) dataset has yielded insightful comparisons. As shown in **Figure** **5**A, DeepLinc demonstrates much higher clustered CCIs in the oligodendrocytes and eL4 cells when compared to STAGUE and COMMOT. Conversely, DeepLinc exhibits fewer CCIs in the eL6‐2 and eL2/3 cells. The neighborhood enrichment analysis between cell types using Squidpy^[^ ^54^ ^]^ also suggests that DeepLinc exhibits a higher degree of clustered CCIs within specific cell types. Similarly, results from two other graph autoencoder‐based methods also display a tendency toward more clustered CCIs (Figure S13, Supporting Information), which is problematic as it fails to accurately capture the interactions between specific cell types. Specifically, CLARIFY underrepresents CCIs involving eL6‐2 cells and eL2/3 cells, and TENET shows limited detection of CCIs in oligodendrocytes.

Next, we examined the correlation between the values in the adjacency matrix and normalized distances. The results show that under various *k* settings, CLARIFY and TENET demonstrate a more discernible pattern of decreasing adjacency values with increasing distances, in contrast to DeepLinc (Figure S14, Supporting Information). However, the adjacency values from all three methods do not show a trend where increasing the *k* value significantly reduces adjacency values for distant CCIs compared to proximal ones. This pattern is uniquely observed in STAGUE (Figure 4C,D), suggesting that it more effectively captures the true spatial distances between cells.

In conclusion, the distribution of inferred CCIs from STAGUE is more uniform and aligns more closely with spatial distances compared to graph autoencoder‐based methods, potentially offering a better reflection of biological priors.

### 2.7 STAGUE Identifies Signature Genes Regulating CCI Networks

Following the approach in DeepLinc,^[^ ^9^ ^]^ we employed STAGUE to identify signature genes regulating the CCI networks. This involved shuffling the expression of one gene at a time across all cells and feeding the altered data into STAGUE, generating a unique cell graph adjacency matrix for each gene. The sensitivity score for each gene was computed to quantify its impact, reflecting changes in the new adjacency matrix relative to the original one from unaltered expression data. These scores shed light on each gene's influence on the CCI network. We ranked all genes in descending order based on their respective sensitivity scores. Subsequently, the Gene Ontology (GO) biological process enrichment analysis^[^ ^55^ ^]^ was performed for genes with high sensitivity scores (signature genes) to find the over‐represented biological processes.

We first analyze the results of the mouse primary visual cortex (V1) and medial prefrontal cortex (BZ5). For V1 (Figure 5B), the signature genes (Figure S15A, Supporting Information) are enriched by multiple biological processes related to CCIs, including chemical synaptic transmission, plasma membrane cell adhesion, neuropeptide signaling, and cell junction formation. The *Ina* gene, which shows the highest sensitivity score, can provide structural support to neurons, affecting synapses' stability and plasticity. Other top ten genes in Figure S15A (Supporting Information) include *Slc7a4* (cationic amino acid transporter, involved in the amino acid transmembrane transport), *Tmem215* (transmembrane protein 215), *Rasgrf2* (Ras guanine nucleotide exchange factor, involved in the activation of MAPK/ERK pathway),^[^ ^56^ ^]^ and *Th* (tyrosine hydroxylase, the rate‐limiting enzyme in the synthesis of catecholamines). For BZ5 (Figure 5C), the signature genes are mainly involved in chemical synaptic transmission, neurotransmitter secretion, neurotransmitter receptor activity, and regulated exocytosis. The highest sensitive gene, *Cplx3*, is involved in regulating SNARE protein complex‐mediated synaptic vesicle fusion. Other top ten genes include *Cdh13* (calcium‐dependent cell adhesion protein), *Cbln4* (secreted protein, involved in the regulation of neurexin signaling), and *Adgrl2* (G‐protein coupled receptor, involved in the regulation of exocytosis) (Figure 15B, Supporting Information).

Our next analysis focuses on the preoptic region of the mouse hypothalamus at Bregma–0.04 mm and –0.09 mm (Figure 5D,E). The signature genes identified at these locations primarily engage in neuropeptide signaling, chemical synaptic transmission, G protein‐coupled receptor signaling, and transmembrane receptor protein tyrosine kinase signaling. At Bregma‐0.04, *Ttyh2* was identified as the most sensitive gene, localized to the plasma membrane and involved in calcium ion signal transduction. Meanwhile, at Bregma–0.09, the most sensitive *Gad1* is responsible for catalyzing the synthesis of the inhibitory neurotransmitter gamma‐aminobutyric acid (GABA). See Figure S15C,D (Supporting Information) for the full list of the top ten sensitive genes.

### 2.8 Systematic Parameter Analyses and Ablation Study of STAGUE

Compared with previous graph contrastive learning‐based methods in spatial clustering, the main difference of STAGUE can be summarized into three parts 1) parameterizing the adjacency matrix to generate learnable graph views, 2) using the feature masking technique for data augmentation, and 3) integrating the NT‐Xent loss and triplet loss to balance the contrast between different views. To verify the effectiveness of these proposed components, we conducted systematic parameter analyses and ablation experiments of STAGUE. Additionally, the effect of the GCN encoders was explored. For these analyses, we used the Real#1 dataset group from the clustering benchmark. The parameter settings were aligned with those used in the clustering benchmark. Unless otherwise specified, only the analyzed parameter was altered during the analyses.

#### 2.8.1 Analysis of $k_{\Gamma }$ and *k*

In this section, we investigate the impact of two hyperparameters: $k_{\Gamma }$, which sketches the raw adjacency matrix Γ, and *k*, which is responsible for determining the cutoff distance *d*_*c*_ for the learned adjacency matrix in Equation (5). For each $k_{\Gamma }$ and *k* value, we run STAGUE five times across all the fourteen datasets in Real#1 and compute the average ARI and AMI.

**Figure 6:**
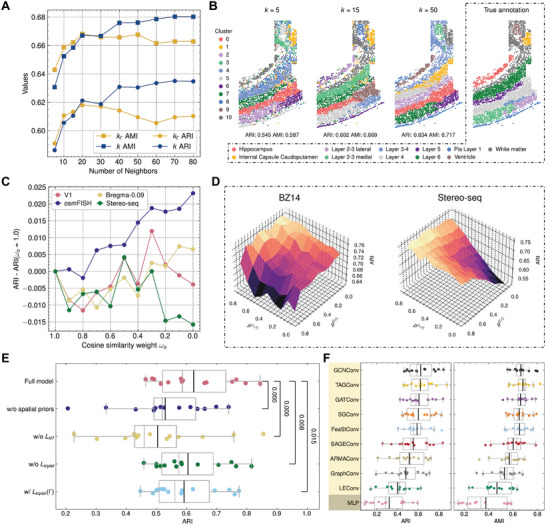
Systematic parameter analyses and ablation study of STAGUE. A) ARI and AMI performance with different $k_{\Gamma }$ and *k* values. Each point represents the mean value of all datasets. For single‐cell data, $k_{\Gamma }$ varies from 5 to 80, while for spot‐level data, the range begins at 6 and proceeds in the same manner. Spot‐level results with $k_{\Gamma }=6$ are shown as $k_{\Gamma }=5$ for clarity. The range of *k* is set between 5 and 80. See Figure S16 (Supporting Information) for the distribution of metrics corresponding to each value of $k_{\Gamma }$ and *k*. B) Clustering results of the osmFISH dataset with different values of *k*. C) ARI performance relative to ω_*o*_ = 1.0 across different ω_*o*_ values with *k* = 15 and γ = 2. D) ARI performance of the BZ14 and Stereo‐seq datasets with different feature masking rates *p* ^(*l*, *r*)^ and *p* ^(+, *r*)^. The values range from 0.0 to 0.8 with a step of 0.1. E) ARI performance of STAGUE with different settings and F) ARI and AMI performance with different GCN encoders and MLP. The *p*‐value was calculated with the one‐sided Wilcoxon signed‐rank test. Each point represents the result of the corresponding method on one dataset. The black center line indicates the mean value across all datasets. Boxplot: center line, the median; upper and lower edges, the interquartile range; whiskers, 1.5 × interquartile range.

We first explore the effect of $k_{\Gamma }$. Note that rather than using the learned adjacency *A*, we employed a fixed Γ to construct the learner view and obtain the learner embeddings. This setting resembles GRACE,^[^ ^57^ ^]^ as both methods employ the raw adjacency matrix and feature matrix to generate two augmented graph views, involving edge‐removing and feature masking. As illustrated in Figure 6A, it is observed that increasing the value of $k_{\Gamma }$ from 5 to 10 substantially improves the performance. However, raising the value to 20 yields only marginal gains, and additional increments lead to a decline in performance. This may be due to the noise increment outpacing the gain in useful information with rising values of $k_{\Gamma }$.

Next, to fairly validate the spatial learner module, experiments were performed with varying *k* values while maintaining a small constant $k_{\Gamma }$. Specifically, $k_{\Gamma }$ is set to 5 for single‐cell‐level data and to 6 for hexagonal‐grid‐like spot‐level data, which is the same setting as the clustering benchmark. As illustrated in Figure 6A, the performance is significantly improved when *k* is increased from 5 to 20. Beyond this point, performance gains become marginal and eventually converge at a *k* value of 80. In the clustering benchmark, *k* is generally set to 15 or 20. Consider the osmFISH dataset from the mouse somatosensory cortex (Figure 6B). Raising *k* from 5 to 15 yields a substantial 10.5% enhancement in ARI. However, further increasing *k* to 50 results in a more modest gain of 5.3%, despite the threefold increase in *k* from 15 to 50. Figure S17 (Supporting Information) demonstrates that for *k* = 50, the learned adjacency values are biased by the spatial decay term in Equation (5), with only 31.6% exceeding 0.5. Conversely, with a smaller *k* value of 5, a majority (71.2%) of values surpass this threshold (Figure S17, Supporting Information). This indicates that with larger *k* values, the influence on the central cell is limited to a small subset of neighbors, which we argue may alleviate the over‐smoothing effect from neighboring cells.

In conclusion, the spatial learner module of STAGUE provides a significant advantage over using a fixed adjacency matrix Γ. By introducing a parameterized adjacency matrix, STAGUE can learn to generate effective graph views for contrastive learning, which in turn facilitates the capture of underlying spatial and gene expression information within the data.

#### 2.8.2 Analysis of ω_*o*_

This section explores the effect of the parameter ω_*o*_ in Equation (5). The role of ω_*o*_ is to balance the cosine similarity with the spatial decay term in the learned adjacency matrix, where a lower value of ω_*o*_ gives more weight to the spatial decay term relative to the cosine similarity. As illustrated in Figure 6C, reducing ω_*o*_ significantly improves the osmFISH dataset's performance. However, the Bregma–0.09, V1, and Stereo‐seq datasets exhibit more modest improvements. This difference could be attributed to the significantly lower gene coverage in osmFISH (only 33 genes) compared to Bregma–0.09, V1, and Stereo‐seq (hundreds to thousands of genes) (Table S1, Supporting Information). Insufficient gene coverage may not yield adequate information for learning cell embeddings, which could impair the modeling of the cosine similarity term. This necessitates a bias toward the spatial decay term. Therefore, it is advisable to increase the weight of the spatial decay term for datasets with low gene coverage. In the clustering benchmark, ω_*o*_ is set to 0.5.

#### 2.8.3 Analysis of Feature Masking Rates

This section investigates the feature masking rates for both the learner view (*p* ^(*l*, *r*)^) and the positive view (*p* ^(+, *r*)^). Note that for the negative view, the feature masking rate *p* ^(−, *r*)^ is set equal to *p* ^(+, *r*)^. Figure 6D illustrates that increasing *p* ^(+, *r*)^ enhances performance for both the mouse medial prefrontal cortex BZ14 and the Stereo‐seq mouse olfactory bulb datasets, albeit with distinct patterns. Specifically, BZ14 shows performance improvement when *p* ^(*l*, *r*)^ decreases, peaking at approximately 0.2. Conversely, Stereo‐seq benefits from an increased *p* ^(*l*, *r*)^, with optimal performance near 0.6. We speculate that the observed phenomenon could be attributed to the different gene coverage levels in the datasets. The lower gene coverage of BZ14 compared to Stereo‐seq (166 vs 3,000) suggests that excessive feature masking in the learner view might hinder the learning of the adjacency matrix. In conclusion, for datasets with high gene coverage, it is recommended to apply larger feature masking rates for both the learner and positive views. Conversely, for datasets with lower gene coverage, a moderate rate should be favored for the learner view. In the clustering benchmark, *p* ^(+, *r*)^ is typically set to 0.9, while *p* ^(*l*, *r*)^ is set lower at 0.6.

#### 2.8.4 Ablation Study

For the ablation study, the following settings of STAGUE are considered: 1) remove the spatial regularization in the learned adjacency matrix, and instead use the kNN algorithm based on cosine similarity to construct the adjacency matrix; 2) remove the NT‐Xent loss $L_{NT}$; 3) remove the triplet loss $L_{triplet}$; 4) use the raw adjacency matrix Γ to generate the negative embeddings instead of using the identity matrix. Figure 6E and Figure S18 (Supporting Information) present the comparison of ARI and AMI for STAGUE under different scenarios, employing all 14 ST datasets in the clustering benchmark. The model was executed five times on each dataset, and the average scores were calculated to ensure robustness in the performance comparison.

Our findings reveal that the exclusion of spatial priors significantly compromises the model's performance. This outcome suggests that even though spatial decay and cutoff distance might reduce certain information by diminishing the influence of distant cells, the benefit of minimizing noise is more significant than this information loss. Eliminating $L_{NT}$ results in a marked decrease in performance, underscoring its importance in the contrast between the learner view and the positive view. A similar trend is also observed with the omission of $L_{triplet}$. This indicates that only using $L_{NT}$ may not be sufficient to help the model discriminate negative samples, and incorporating an explicit negative view along with the triplet loss can enhance the model performance. However, it is important to note that employing the raw adjacency matrix Γ for generating negative embeddings would even impair the performance compared to using $L_{NT}$ alone, i.e., w/ $L_{triplet}\left(\Gamma \right)$ vs w/o $L_{triplet}$. This suggests that using the topological information in Γ to encode negative embeddings could result in the blurring of certain distinctive characteristics of negative samples.

#### 2.8.5 GCN Encoder Types

In this study, we utilized the GCNConv model^[^ ^28^ ^]^ as the default GCN encoder within the STAGUE framework. This section provides a comparative analysis of the performance across nine different GCN encoders. Additionally, we employed a two‐layer MultiLayer Perceptron (MLP) with ReLU activation as a baseline for comparison.

Figure 6F demonstrates that the GCNConv and TAGConv^[^ ^58^ ^]^ models outperform other GCN encoders, achieving average ARI/AMI scores of 0.624/0.665 and 0.620/0.681, respectively. In contrast, the MLP model demonstrates markedly inferior performance, yielding an ARI of 0.319 and an AMI of 0.378. These findings suggest that for STAGUE, the GCN encoder outperforms the MLP by effectively capturing and utilizing the spatial relationships inherent in the data, with the GCNConv and TAGConv models outperforming other GCN encoder variants.

## 3 Discussion

Spatial transcriptomic (ST) technologies, which preserve spatial information during gene expression profiling, provide a unique opportunity for the identification of spatially coherent clusters and the elucidation of cell‐cell interactions (CCIs) with improved biological precision. Since the pioneering studies in spatial domain identification around 2021, including tools like BayesSpace,^[^ ^59^ ^]^ SpaGCN,^[^ ^4^ ^]^ etc., there has been significant interest in adopting cutting‐edge unsupervised graph representation learning (UGRL) techniques for spatial clustering^[^ ^29^ ^]^ and CCI inference.^[^ ^9^, ^10^, ^11^ ^]^ Despite the proliferation of numerous methods in this field, many suffer from inherent limitations primarily because they rely solely on spatial information to define graph structure, which can be insufficient and challenging. This study introduces STAGUE, a framework that, for the first time, introduces graph structure learning into unsupervised representation learning of ST data. STAGUE generates low‐dimensional cell embeddings and a learned adjacency matrix for the cell graph, capturing the statistical dependencies between cells. The cell embeddings can be used for multiple downstream applications such as clustering, visualization, and trajectory inference, whereas the cell graph adjacency matrix can be used to discern novel CCIs. In this way, STAGUE seamlessly fuses the tasks of spatial clustering and CCI inference within a unified framework.

Benchmarking analysis of spatial clustering illustrates that STAGUE outperforms other methods, achieving the best overall performance across two real and two simulated dataset groups, covering a total of 86 datasets. Moreover, it exhibits greater robustness against variations in the sequencing quality of the input data. Additionally, STAGUE exhibits a remarkable capacity to discern heterogeneity in human breast cancer tissues, notably revealing the activation of epithelial‐to‐mesenchymal transition and PI3K/AKT signaling in specific sub‐regions. The scalability analysis demonstrates that STAGUE is capable of processing 87 305 cells within 2 min using a standard device equipped with 64 GB of CPU memory and 24 GB of GPU memory, highlighting its suitability for large datasets. Further analysis of STAGUE's learned adjacency matrix reveals that it effectively captures a key characteristic of CCIs–the selective attenuation of interaction strength with increasing distance, a feature underrepresented by existing graph autoencoder‐based CCI inference methods. Finally, the systematic parameter analyses and ablation experiments have verified the effectiveness of the proposed components in STAGUE.

The benchmarking results reveal that spatially informed methods encounter challenges in the Real#2 dataset group, characterized by small, discrete domains. Notably, the non‐spatial baseline, Scanpy, achieves the highest AMI score among all methods, while SiGra shows improved performance by incorporating image data. Based on these findings, we propose two potential directions for refining STAGUE: 1) *Enhancing information aggregation in GNN models by incorporating biological priors*. In GNNs, the mechanism of aggregating information from neighboring cells can sometimes obscure the differences between central cells that display distinct gene expressions but are located in similar environments. This is particularly problematic in datasets with small and discrete domains, where it can lead to incorrect assignments of tissue domains. Currently, STAGUE relies solely on spatial information and gene expression data to model CCIs within the adjacency matrix, guiding the aggregation process. Here, we anticipate that the integration of additional biological priors, such as ligand‐receptor pairs and intracellular signaling pathways,^[^ ^60^ ^]^ can significantly refine the adjacency matrix and enhance the aggregation process. CLARIFY explores this concept by transforming the cell graph into a gene graph, where each cell is represented as a gene regulatory network. However, to manage computational demands, subgraph sampling algorithms may be utilized.^[^ ^61^ ^]^ 2) *Integration of additional tissue morphology information for improved clustering*. Our analysis indicates that SiGra performs better on Real#2 by utilizing image data. This improvement underscores the benefits of incorporating detailed tissue morphology into spatial clustering methods. Approaches such as stLearn, SpaGCN, and SiGra, which consider this integration, together with advancements in the image segmentation field,^[^ ^62^ ^]^ provide valuable insights and methodologies for delineating complex spatial heterogeneity.

## 4 Experimental Section

### Datasets—Real#1

The Real#1 dataset group consists of 14 datasets from five ST platforms (see Table S1 for details, Supporting Information). The STARmap datasets^[^ ^1^ ^]^ measure the mouse primary visual cortex (V1) with 1207 cells and 1020 genes (https://www.starmapresources.com/data), and three mouse medial prefrontal cortex (mPFC) sections, i.e., BZ5, BZ9, and BZ14, all of which with roughly 1050 cells and 166 genes (https://github.com/zhengli09/BASS‐Analysis). The MERFISH datasets^[^ ^63^ ^]^ contain two selected mouse hypothalamic preoptic sections at Bregma–0.04 mm and Bregma–0.09 mm, profiling 5488 and 5557 cells with 155 genes (https://github.com/zhengli09/BASS‐Analysis). The osmFISH dataset^[^ ^64^ ^]^ includes 4839 cells and 33 genes from the mouse somatosensory cortex (http://linnarssonlab.org/osmFISH/availability). The processed Stereo‐seq dataset^[^ ^40^ ^]^ encompasses 10 000 cells and 26 145 genes from the mouse olfactory bulb (https://github.com/acheng416/Benchmark‐CTCM‐ST). The spot‐level 10x Visium datasets measure the human dorsolateral prefrontal cortex (DLPFC).^[^ ^2^ ^]^ Specifically, six slices with different spatial domain patterns were selected, profiling 3888–4788 spots and 33 538 genes (http://spatial.libd.org/spatialLIBD).

### Datasets—Real#2

The Real#2 dataset group, available at http://sdmbench.drai.cn/ , comprises 16 datasets. This collection includes 1) seven datasets of HER2‐positive breast tumors using spatial transcriptomics technology^[^ ^65^ ^]^ (167–659 spots, ∼15 000 genes), 2) seven datasets of liver tissues using 10x Visium^[^ ^66^ ^]^ (1121–2002 spots, ∼30 000 genes), and 3) two datasets of human pancreatic ductal adenocarcinomas using spatial transcriptomics technology^[^ ^67^ ^]^ (428 and 224 spots, 19 738 genes). See Table S2 (Supporting Information) for details.

### Datasets—Simulated#1

The Simulated#1 dataset group contains 28 datasets generated using SRTsim^[^ ^30^ ^]^ (https://github.com/xzhoulab/SRTsim). The Real#1 group served as the reference for conducting reference‐based simulations. To enhance the diversity of the simulations, both tissue‐based and domain‐specific approaches were considered.

### Datasets—Simulated#2

The Simulated#2 dataset group comprises 28 datasets generated using scSpace^[^ ^31^ ^]^ (https://github.com/ZJUFanLab/scSpace). The *sim_data_construct()* method was utilized for simulations involving 1000 cells, with varying numbers of cell clusters and subclusters. Specifically, all the cell cluster configurations used in scSpace were employed, including several cell clusters and 2–10 refined cell subclusters (Table S3, Supporting Information).

### Datasets—BRCA dataset

The BRCA Block A Section 1 sample used in the clustering result visualization was sourced from the 10x Genomics database, available at https://www.10xgenomics.com. The dataset comprises 3798 spots and includes 36 601 genes. The number of clusters was changed to predict and analyze the heterogeneity within cancerous and paracancerous tissues.

### STAGUE—Feature Processing

From ST data, the gene expression count matrix $X^{o}\in R^{N\times F_{g}}$ can be obtained, where *N* is the number of cells and *F*_*g*_ denotes the number of genes. Additionally, the cells' spatial coordinates ${\left\{c_{i}\right\}}_{i=1}^{N}$ can be acquired, with each coordinate **c**_*i*_ typically in $R^{2}$. Note that ST data can be at single‐cell resolution or a more coarse spot‐level resolution, where a spot comprises multiple cells. The following algorithm introduction interchangeably uses “cell” to refer to a “spot.” For datasets containing over 3000 genes, the Seurat v3^[^ ^68^ ^]^ flavor of Scanpy package^[^ ^69^ ^]^ was first utilized to identify 3000 highly variable genes.

Due to the varying scales, sparsity, and noise in the count values for each cell, a preprocessing step was applied to convert the raw count matrix into a more structured feature matrix $X\in R^{N\times F}$,

(1) $$\begin{array}{cc} X & =f_{p}^{2}\left(f_{p}^{1}\left(X^{o}\right)\right) \end{array}$$

(2) $$\begin{array}{cc} f_{p}^{1}\left(X_{ij}^{o}\right) & =\ln\left(\text{median}\left(X^{o}\right)\frac{X_{ij}^{o}}{\sum_{j=1}^{F_{g}}X_{ij}^{o}}+1\right) \end{array}$$

where median(*X* ^*o*^) is the median of total counts for cells; $f_{p}^{2}\left(\cdot \right)$ represents a MultiLayer Perceptron (MLP) with two linear layers, each followed by a ReLU activation, and ending with a BatchNorm1d layer^[^ ^70^ ^]^ for output normalization. Next, the k‐nearest neighbors (kNN) algorithm was employed to construct a raw adjacency matrix Γ = {0, 1}^*N* × *N*^ based on the Euclidean distance between cell pairs, i.e., *d*_*ij*_ = ||**c**_*i*_ − **c**_*j*_||_2_. This step entails a *k*_Γ_ to determine the number of neighbors for each cell. This preliminary adjacency matrix Γ serves as the basis for generating both the learner and positive views of the input ST data. To reduce the noise when sketching Γ, we adopt the strategies in DeepLinc^[^ ^9^ ^]^ and CLARIFY,^[^ ^10^ ^]^ setting *k*_Γ_ to a low value, e.g., 5.

### STAGUE—Spatial Learner View

It was aimed to devise a spatial learner module capable of learning a parameterized adjacency matrix from the feature matrix and spatial information. Through optimization, this module is designed to craft the learner view effectively.

According to the network homophily assumption that edges are more likely to connect similar nodes, many metric‐based approaches have been proposed to compute the edge weights, including Gaussian,^[^ ^71^ ^]^ inner‐product,^[^ ^72^ ^]^ and cosine similarity^[^ ^73^, ^74^ ^]^ kernels. This framework offers flexibility in selecting kernels. Here, simplicity was opted and the cosine similarity kernel was adopted with a uniform weight vector for the node features,

(3) $$A_{ij}^{o}=\sigma \left(\cos\left(h_{i}^{o},h_{j}^{o}\right)\right)$$

where cos (·) is cosine similarity, σ(·) = max(0, ·) is the ReLU nonlinearity, and $h_{i}^{o}$ and $h_{j}^{o}$ are the features of two corresponding input nodes. These node features, denoted as $H^{o}={\left\{h_{i}^{o}\right\}}_{i=1}^{N}$, are derived from the final layer's node embeddings of a two‐layer Graph Convolutional Network (GCN) model,^[^ ^28^ ^]^ *f*_*o*_(·):

(4) $$H^{o}=f_{o}\left(X,\Gamma \right)=\tilde{\Gamma }\sigma \left(\tilde{\Gamma }XW_{1}^{o}\right)W_{2}^{o}$$

where $\tilde{\Gamma }={\hat{D}}_{\Gamma }^{-\frac{1}{2}}\hat{\Gamma }{\hat{D}}_{\Gamma }^{-\frac{1}{2}}$ represents the normalized adjacency matrix, with $\hat{\Gamma }=\Gamma +I$ and ${\hat{D}}_{\Gamma }$ as its degree matrix; $W_{1}^{o}$ and $W_{2}^{o}$ are trainable parameters of the encoder layer.

The sketched adjacency matrix *A* ^*o*^ from cosine similarity is often dense and needs to be regularized to satisfy sparsity. Popular methods include penalizing the ℓ_0_‐norm of the adjacency matrix^[^ ^75^ ^]^ or using the kNN algorithm to retain the top‐k values for each node and set the rest to zero.^[^ ^25^ ^]^ Here, the focus is on using the spatial information prior to regularize the learned adjacency matrix. Specifically, the following equation is applied to refine *A* ^*o*^,

(5) $$A_{ij}^{\prime }=\left\{\begin{array}{ccc} & \omega_{o}A_{ij}^{o}+\left(1-\omega_{o}\right)e^{-\gamma {\left(\frac{d_{ij}}{d_{c}}\right)}^{2}}, & 0<d_{ij}\leq d_{c}\\ & 0, & \text{otherwise}, \end{array}\right.$$

where ω_*o*_, γ, and *d*_*c*_ are hyperparameters. The term ω_*o*_ balances the cosine similarity and exponentially spatial decay, γ controls the decay rate, and *d*_*c*_ defines a cutoff distance for ignoring long‐range node connections. Kindly note that a larger *d*_*c*_ is permissible here to capture a broader neighborhood. This is made possible by the noise regulation effect provided by both the original adjacency matrix $A_{ij}^{o}$ and the spatial decay term. Here, *d*_*c*_ is set to the median value of the distances between each cell and its *k*‐th nearest neighbor, where *k* can be set to a larger number, such as 20. Finally, the edge weights in *A*′ are confined to [0,1] through the symmetric normalization, producing $A=D^{\prime -\frac{1}{2}}A^{\prime }D^{\prime -\frac{1}{2}}$, with *D*′ as the degree matrix of *A*′.

Two data augmentation strategies that produce an augmented version of feature matrix $\;\bar{\;X}$ and adjacency matrix $\;\bar{\;A}$ were employed to create the learner view. Similar augmentation methods are adopted to develop positive and negative views. For the sake of clarity and readability, a detailed elaboration is provided in the subsequent Data Augmentation Section. Finally, a two‐layer GCN encoder *f*_*l*_(·) was utilized with the same structure as *f*_*o*_(·) to generate the learner embeddings,

(6) $$H=f_{l}\left(\;\bar{\;X},\;\bar{\;A}\right)$$

### STAGUE—Positive View

Existing works generally incorporate the structural information into the generation of positive embeddings, such as DGI,^[^ ^21^ ^]^ GRACE^[^ ^57^ ^]^ and GIC;^[^ ^76^ ^]^ here, this convention was followed to encode the positive view,

(7) $$H^{+}=f_{+}\left(\;\bar{\;X},\;\bar{\;\Gamma }\right)$$

where *f*_+_(·) is a two‐layer GCN encoder sharing weights with *f*_*l*_(·), and $\;\bar{\;X}$ and $\;\bar{\;\Gamma }$ are the augmented feature and adjacency matrix of the positive view, respectively.

Inspired by SGC‐LL^[^ ^77^ ^]^ and SUBLIME,^[^ ^25^ ^]^ it was hypothesized that the learned adjacency matrix *A* represents a subtle deviation from the original noisy Γ, an adjustment that could potentially enhance the learning process for the positive view. Consequently, a connection between Γ and *A* was implemented to update Γ iteratively,

(8) $$\Gamma \leftarrow \omega_{\Gamma }\Gamma +\left(1-\omega_{\Gamma }\right)A$$

where $\omega_{\Gamma }$ is the conservation rate of the original Γ and fixed at 0.999.

### STAGUE—Negative View

The implementation of hard negative sampling was considered to establish an explicit negative view. Specifically, a row‐wise random shuffle was first applied to the feature matrix *X*, yielding the negative features *X* ^−^; it was further encoded using the identity matrix as the adjacency matrix,

(9) $$H^{-}=f_{-}\left(\;{\bar{\;X}}^{-},\;\bar{\;I}\right)$$

where *f*_−_(·) is a two‐layer GCN encoder sharing weights with *f*_*l*_(·), and $\;{\bar{\;X}}^{-}$ and $\;\bar{\;I}$ are the augmented feature and adjacency matrix of the negative view, respectively. This process can be interpreted as directly passing the augmented feature matrix through a two‐layer MLP connected by a ReLU activation function and sharing weight parameters with *f*_*l*_(·).

Note that popular methods generally encode the negative embedding using the original adjacency matrix.^[^ ^19^, ^20^, ^21^ ^]^ This approach can be interpreted as encoding a negative graph that is isomorphic to the positive one, thereby maintaining structural correspondence between the two. Here, it was argued that using the structural information in Γ to encode the shuffled matrix *X* ^−^ could potentially lead to an undesirable smoothing effect, thereby diminishing distinctive features intrinsic to the negative samples.

### STAGUE—Data Augmentation

Recent research has elucidated that both feature augmentation and graph topology augmentation are advantageous for enhancing the performance of GCL.^[^ ^25^, ^57^, ^78^ ^]^ An empirical study of GCL^[^ ^15^ ^]^ has highlighted two particular augmentation strategies: 1) randomly masking a fraction of feature dimensions with zeros and 2) removing edges to build sparser graph views. In this study, these two data augmentation approaches were jointly used to process three views,

(10) $$\begin{array}{cc} H & =f_{l}\left(X⊙M^{\left(l,r\right)},A⊙M^{\left(l,e\right)}\right),\\ H^{+} & =f_{+}\left(X⊙M^{\left(+,r\right)},\Gamma ⊙M^{\left(+,e\right)}\right),\\ H^{-} & =f_{-}\left(X^{-}⊙M^{\left(-,r\right)},I\right) \end{array}$$

Here, $M^{\left(\cdot ,r\right)}\in R^{N\times F}$ denotes a masking matrix applied to the feature matrix. This matrix is constructed by initially generating a binary mask vector **m** ^(·, *r*)^ ∈ {0, 1}^*F*^, where each element is independently drawn from a Bernoulli distribution $B(1-p^{\left(\cdot ,r\right)})$, with *p* ^(·, *r*)^ representing the probability of removing a feature dimension. The vector **m** ^(·, *r*)^ is then replicated *N* times and transposed to form the feature masking matrix *M* ^(·, *r*)^:

(11) $$M^{\left(\cdot ,r\right)}={\left[m^{\left(\cdot ,r\right)};\text{…};m^{\left(\cdot ,r\right)}\right]}^{⊤}$$

where [· ; ·] represents the concatenation operation. Similarly, *M* ^(·, *e*)^ ∈ {0, 1}^*N* × *N*^ is a binary masking matrix for the adjacency matrix. Each entry of it, $M_{ij}^{\left(\cdot ,e\right)}$, is independently sampled from a Bernoulli distribution $B(1-p^{\left(\cdot ,e\right)})$, with *p* ^(·, *e*)^ representing the probability of an edge being removed. All edges in the identity matrix were maintained intact for generating *H* ^−^, as the GCN encoder's self‐loop insertion and edge weight normalization mechanisms ensure the outcome remains unaffected. An identical feature masking rate was employed for both positive and negative embeddings, i.e., *p* ^(+, *r*)^ = *p* ^(−, *r*)^.

### STAGUE—Joint Contrastive Objectives

Given the embeddings corresponding to the learner, positive and negative views, they were projected into a common space for contrastive loss evaluation using a shared projection head ϕ(·). For illustration, consider the learner embedding as an example:

(12) $$Z=\phi \left(H\right)=\sigma \left(HW_{1}^{\phi }\right)W_{2}^{\phi }$$

where $W_{1}^{\phi }$ and $W_{2}^{\phi }$ are trainable parameters. Similarly, *Z* ^+^ and *Z* ^−^ were derived from the positive and negative embeddings.

Contrastive learning aims to make positive pairs (i.e., *Z* and *Z* ^+^) close while keeping negative pairs (i.e., *Z* and *Z* ^−^) far apart. To achieve this, the selection of contrastive objectives is very essential. Here, a symmetrically normalized temperature‐scaled cross‐entropy (NT‐Xent) loss^[^ ^25^, ^27^ ^]^ was utilized to maximize the similarity between the corresponding node pairs in *Z* and *Z* ^+^,

(13) $$\begin{array}{cc} L_{NT} & =\frac{1}{2N}\sum_{i=1}^{N}\left[\ell \left(z_{i},z_{i}^{+}\right)+\ell \left(z_{i}^{+},z_{i}\right)\right] \end{array}$$

(14) $$\begin{array}{cc} \ell (z_{i},z_{i}^{+}) & =-\log\frac{\exp(\cos\left(z_{i},z_{i}^{+}\right)/\tau )}{\sum_{k=1}^{N}\exp\left(\cos\left(z_{i},z_{k}^{+}\right)/\tau \right)} \end{array}$$

where τ denotes a temperature parameter. The value of τ is supposed to be on the small side, such as 0.3, to make the loss curve steeper and facilitate comparisons.

Recent studies have shown that the $L_{NT}$‐like loss inherently performs hard negative sampling. However, this method indiscriminately distances **z**_*i*_ from every other $z_{k}^{+}$ where *k* ≠ *i* without considering their possible intra‐class relationships.^[^ ^15^ ^]^ To address this, we incorporate a triplet loss to reduce the intra‐class variation and amplify the inter‐class variation,

(15) $$L_{triplet}=\frac{1}{N}\sum_{i=1}^{N}\max\left(d\left(z_{i},z_{i}^{+}\right)-d\left(z_{i},z_{i}^{-}\right)+\varepsilon ,0\right)$$

where *d*(·) is the ℓ_2_‐norm distance and ϵ is a small non‐negative margin, such as 0.5, that ensures a “safe” distance between positive and negative pairs. Finally, the NT‐Xent loss and triplet loss are combined as follows:

(16) $$L=L_{NT}+\omega_{t}L_{triplet}$$

where ω_*t*_ is the weight of $L_{triplet}$ to control the influence of hard negative samples.

### STAGUE—Scalability Extension

Two computationally intensive tasks within STAGUE exhibit $O(n^{2})$ complexity in space and time: 1) calculating pairwise cosine similarities, and 2) computing the NT‐Xent loss. To enhance the method's scalability on large datasets, STAGUE addresses the first issue by sparsifying the cosine similarity matrix using the cutoff distance *d*_*c*_ between cells. To tackle the second issue, the NT‐Xent loss is calculated on a mini‐batch of samples rather than on all nodes, thereby reducing memory demands.

### Evaluation Settings

For the spatial clustering benchmark, the comparison methods include one popular single‐cell RNA‐sequencing data analysis pipeline (i.e., Scanpy^[^ ^69^ ^]^), four unsupervised learning algorithms without using graph contrastive learning (GCL) (i.e., SpaGCN,^[^ ^4^ ^]^ DeepLinc,^[^ ^9^ ^]^ SEDR,^[^ ^5^ ^]^ and STAGATE^[^ ^7^ ^]^), four GCL‐based unsupervised algorithms (i.e., CCST,^[^ ^17^ ^]^ SpaceFlow,^[^ ^19^ ^]^ SCAN‐IT,^[^ ^18^ ^]^ and GraphST^[^ ^20^ ^]^), and two methods that use tissue morphology information (i.e., stLearn^[^ ^79^ ^]^ and SiGra^[^ ^35^ ^]^). Additionally, four state‐of‐the‐art statistical learning methods were included for comparison, including BayesSpace,^[^ ^59^ ^]^ SpatialPCA,^[^ ^36^ ^]^ NSF,^[^ ^80^ ^]^ and BASS.^[^ ^34^ ^]^ The comparison methods were executed on all datasets using their respective recommended hyperparameter values. Similarly, a set of empirical hyperparameter values was used for STAGUE, which may not represent the optimal setting for each individual dataset, as indicated in the Parameter Analyses Section. To mitigate the impact of randomness and ensure a fair comparison, all deep learning methods were executed five times using the same set of random seeds, ranging from 0 to 4, and the average performance was reported. The classic k‐means algorithm was utilized as the default clustering algorithm. See Supporting Information for the detailed description of the parameters and experimental settings for STAGUE and comparison methods.

For comparison in CCI inference, the adjacency matrix generated by STAGUE was evaluated against the reconstructed adjacency matrices from three graph autoencoder‐based methods: DeepLinc, CLARIFY, and TENET. The results of COMMOT^[^ ^53^ ^]^ were used as a reference to facilitate the comparison between STAGUE and autoencoder‐based methods. The cutoff distance utilized for all methods was set to identical, based on the median distance from each cell to its tenth nearest neighbor. For COMMOT, a threshold of 0.2 was used to establish the edges. To ensure a fair comparison between DeepLinc, CLARIFY, TENET and STAGUE, given the differences in their edge value distributions, the threshold for the learned adjacency was carefully adjusted to maintain an edge count similar to that of COMMOT.

### Clustering Metrics

To evaluate the clustering results, two commonly used metrics, namely Adjusted Rand Index (ARI)^[^ ^32^ ^]^ and Adjusted Mutual Information (AMI),^[^ ^33^ ^]^ were employed to measure the similarity between the true annotations and the predicted labels.

Given a set of *N* cells or spots from an ST dataset, the true annotation partition is defined as *U* = {*U*_1_, …, *U*_*c*_} and the predicted label partition as *V* = {*V*_1_, …, *V*_*c*_}, where *c* represents the number of clusters in the true annotations. The ARI is defined as:

(17) ARI=∑ijnij2−∑iai2∑jbj2/N212∑iai2+∑jbj2−∑iai2∑jbj2/N2

where *a*_*i*_ = |*U*_*i*_|, *b*_*j*_ = |*V*_*j*_|, and *n*_*ij*_ represents the number of cells or spots in common between *U*_*i*_ and *V*_*j*_: |*U*_*i*_∩*V*_*j*_|. The AMI is defined as:

(18) $$AMI=\frac{MI(U,V)-E[MI(U,V)]}{\text{mean}(H(U),H(V))-E[MI(U,V)]}$$

where *H*(·) is the entropy, *MI*(·) denotes the mutual information, *E*[*MI*(·)] is the expected mutual information, and mean(·) refers to some generalized mean, such as the geometric or arithmetic mean.

The scikit‐learn library was utilized to calculate these metrics,^[^ ^81^ ^]^ and the arithmetic mean was applied for the AMI. ARI and AMI values near zero suggest that the partitions are largely independent, whereas values approaching one indicate a strong agreement.

### Scalability Analysis

For all methods, the input slices are combined into a larger dataset using the *concat()* method of anndata,^[^ ^82^ ^]^ and this preprocessing step does not count toward the time cost of the method. All experiments were carried out on an Ubuntu 20.04.6 server equipped with 64 GB of RAM and a single NVIDIA RTX 4090 GPU with 24 GB of memory.

### Differential Expression Analysis and Pathway Enrichment Analysis

In the clustering result visualization section for the BRCA dataset, we used the Wilcoxon rank‐sum test in Scanpy^[^ ^69^ ^]^ to find differentially expressed genes (DEGs), all of which exhibited adjusted *p*‐values below 1e‐5. The “KEGG_2021_Human” library^[^ ^83^ ^]^ was used to perform the pathway enrichment analysis through the Enrichr API of GSEAPY.^[^ ^84^ ^]^ Adjusted *p*‐values were reported for the enriched pathways.

### Analysis of the Rectifying Effect of Gene Expression on Spatial Constraints

To identify spatial decay‐violating CCIs, we defined a low threshold at 0.7 × exp (·) for weaker CCIs and a high threshold at 0.7 × exp (·) + 0.3 for stronger CCIs. Neighboring cells within these CCIs were categorized into stronger and weaker groups. The Wilcoxon rank‐sum test in Scanpy was employed to identify differentially expressed genes (DEGs) between these groups, reporting adjusted *p*‐values. Only cells displaying varying CCI strengths with their neighbors were included in the DEG analysis. Those that consistently demonstrated either stronger or weaker interactions with all their neighbors were excluded. Furthermore, certain neighboring cells exhibited stronger CCIs with specific central cells, while demonstrating weaker CCIs with others. These were included in both stronger and weaker groups to enhance the robustness of the DEG analysis.

## Conflict of Interest

The authors declare no conflict of interest.

## Supporting information

Supporting Information

## Data Availability

All data utilized in this study are publicly available, as described in Experimental Section. The source code of STAGUE and related analysis scripts are available at https://github.com/deepomicslab/STAGUE.
