# Supplementary material for: Spatially Informed Graph Structure Learning Extracts Insights from Spatial Transcriptomics
Source: Adv Sci (Weinh). 2024 Oct 9;11(45):2403572. doi: 10.1002/advs.202403572 (PMC11615819; doi:10.1002/advs.202403572)
Supplement: Supplementary file 1 — Supporting Information [file ADVS-11-2403572-s001.pdf]

ADVANCED SCIENCE
Open Access

Supporting Information

for *Adv. Sci.*, DOI 10.1002/advs.202403572

Spatially Informed Graph Structure Learning Extracts Insights from Spatial Transcriptomics

*Wan Nie, Yingying Yu, Xueying Wang, Ruohan Wang and Shuai Cheng Li**

SUPPORTING INFORMATION

Spatially Informed Graph Structure Learning Extracts Insights from Spatial Transcriptomics

Wan Nie[1,†], Yingying Yu[1,†], Xueying Wang[1,2], Ruohan Wang[1], and Shuai Cheng Li[1,⋆]

[1]Department of Computer Science, City University of Hong Kong, Hong Kong SAR, China
[2]City University of Hong Kong (Dongguan), Dongguan, 523000, China
[†]Contributed equally
[⋆]Corresponding author: shuaicli@cityu.edu.hk

Contents

1 Supplementary Notes **2**

2 Supplementary Tables **5**

3 Supplementary Figures **8**

1 Supplementary Notes

Experimental settings for the spatial clustering benchmark

Scanpy. Scanpy was used as a baseline model to show the advantage of considering spatial information. We employed a basic pipeline for the clustering, including count normalization, log transformation, selection of highly variable genes, and principal component analysis (PCA). The top 30 principal components (PCs) were used to calculate the neighborhood graph. A binary search method was used to search for an appropriate resolution for the Louvain clustering algorithm to obtain the same number of clusters as the ground truth.

BayesSpace. BayesSpace applies a Bayesian statistical method for clustering, which uses spatial information to encourage proximal spots to belong to the same cluster. We referenced the pipeline and parameters in the BayesSpace vignette (`https://www.ezstatconsulting.com/BayesSpace/articles/BayesSpace.html`). Specifically, we used the top 15 PCs and 2,000 highly variable genes for data preprocessing. We set the "platform" parameter to "ST" for single-cell resolution datasets and to "Visium" for DLPFC datasets. In all datasets, we configured the spatialCluster function with the "nrep" parameter set to 1000 and the "burn.in" parameter set to 10.

SpatialPCA. SpatialPCA builds on principal component analysis to infer spatially informed cell representations followed by Walktrap or Louvain clustering. We followed the SpatialPCA tutorial website (`https://lulushang.org/SpatialPCA_Tutorial/`). Gaussian kernels were used to model the spatial pattern. The type of bandwidth to be used in Gaussian kernel was set to "SJ". For estimating the loading matrix and the spatial PCs, the low-rank approximation on the kernel matrix was set to "False", and the number of spatial PCs was set to 20. Louvain algorithm was used on the spatial PCs for the clustering of all datasets.

NSF. NSF is based on transformed Gaussian processes to infer spatially informed cell representations followed by the Leiden clustering algorithm [1]. We followed the tutorial in the NSF GitHub repository (`https://github.com/willtownes/nsf-paper`). The number of top PCs was established at 16. Each dataset was randomly divided into a training set, comprising 95% of the observations, and a validation set, accounting for the remaining 5% of the observations. Poisson likelihood was used for all single-cell level data, while Gaussian likelihood was used for the remaining spot-level data.

BASS. BASS is a cell type clustering and spatial domain detection method designed for multi-scale and multi-sample analysis of spatial transcriptomics data. It employs a Bayesian hierarchical modeling approach to conduct clustering analysis. The default parameters provided in the vignette were employed for spatial clustering (`https://zhengli09.github.io/BASS-Analysis/`). Specifically, the top 20 PCs were used for all datasets. For the spot-level dataset, we selected 3,000 highly variable genes for data preprocessing. The "scaleFeature" parameter was set to False, and clustering was initialized using mclust [2]. The number of cell types was maintained at the default value of 20, with the "nsample" parameter set to 10,000. For other datasets, k-means was used for clustering initialization, the number of cell types from the original dataset was retained, and the "scaleFeature" parameter was set to True.

SpaGCN. SpaGCN utilizes a graph encoder to smooth the cell representations and employs DEC [3] to refine initial raw cell clusters iteratively. The non-H&E version was used in this study. We followed the tutorial in the SpaGCN GitHub repository (`https://github.com/jianhuupenn/SpaGCN`). In particular, the number of PCs was set to 50, and the parameter "histology" was set to "False". The model was trained for 200 epochs with the learning rate set to 0.05. We ran the model five times with random seeds {0, 1, 2, 3, 4} and computed the average ARI and AMI performance.

DeepLinc. DeepLinc leverages an adversarially regularized VGAE [4] to learn the latent cell embeddings and uses k-means for clustering. We followed the tutorial in the DeepLinc GitHub repository (`https://github.com/xryanglab/DeepLinc`). The default parameters were used while we raised the number of training epochs to 300. To maximize the model's learning capacity, we allocated the entire dataset for training by setting the test set ratio to zero. For the spatial clustering task, we applied k-means five times to the latent embedding from each epoch using random seeds 0 to 4 and computed the average performance. The best ARI and the corresponding AMI across all epochs were recorded. Furthermore, we ran the model five times with random seeds from 0 to 4 and reported the average performance.

stLearn. stLearn employs the Spatial Morphological gene Expression (stSME) imputation method to integrate spatial location and tissue morphology information from H&E tissue images, adjusting gene expression values across spots. It uses the k-means algorithm for clustering purposes. We followed the stLearn clustering tutorial website (https://stlearn.readthedocs.io/en/latest/tutorials/stSME_clustering.html). The top 15 PCs were used for the clustering of all datasets.

SEDR. SEDR employs a variational graph autoencoder along with a masked self-supervised learning framework to extract latent representations from ST data and uses mclust for clustering. We followed the tutorial in the SEDR GitHub repository (https://sedr.readthedocs.io/en/latest/Tutorial1_Clustering.html). For datasets with over 2,000 genes, the top 2,000 highly variable genes were selected. Clustering was performed using the top 200 PCs; however, for datasets containing fewer than 200 genes, all genes were used in the analysis.

SCAN-IT. SCAN-IT utilizes the DGI framework [5] to derive low-dimensional embeddings for cells, which are subsequently clustered using the k-means algorithm. We followed the recommended parameters in the SCAN-IT repository (https://github.com/zcang/SCAN-IT). Specifically, the number of Self-Organizing Map nodes was set to five. For datasets containing over 3,000 genes, we selected the top 3,000 highly variable genes for data processing. Additionally, we utilized the "alpha shape" mode to construct the spatial adjacency graph, setting the "knn_n_neighbors" parameter to 5 and the "alpha_n_layer" to 2. The hidden size was set to 30, and the model was trained for 500 epochs with a learning rate of 0.01.

CCST. CCST builds upon the DGI framework to generate low-dimensional cell embeddings, which are then clustered using the k-means++ algorithm. We adhered to the instructions provided in the CCST GitHub repository (https://github.com/xiaoyeye/CCST). For datasets with more than 200 genes, the top 200 PCs were utilized for training. Additionally, the parameter λ_i is set to 0.3 for the DGI model whose dimension of each hidden layer is 256. This model is trained for 5,000 epochs with a learning rate of 0.000001.

SiGra. SiGra enhances sparse and noisy transcriptomics data by leveraging imaging information to derive cell embeddings for Leiden clustering. It employs hybrid graph transformers to handle multimodal data, including multichannel images of cells and their niches. We followed the recommended parameters in the SiGra tutorial (https://github.com/QSong-github/SiGra). For the DLPFC dataset, the mclust algorithm was employed for clustering. For datasets containing image data, we utilized the *train_img()* method to train the model, while for non-image datasets, we employed the *train_nano_fov()* method. The training was carried out across 2,000 epochs with a learning rate set to 0.001.

STAGATE. STAGATE employs a graph attention auto-encoder (GAT) [6] and utilizes mclust for clustering. We utilized the PyTorch Geometric version of STAGATE (https://github.com/QIFEIDKN/STAGATE_pyG). The default parameters were used. In particular, the top 3,000 highly variable genes were first selected, followed by count normalization and log transformation. The model was trained for 500 epochs with a learning rate of 0.001.

SpaceFlow. SpaceFlow is based on the DGI framework to infer low-dimensional embeddings for cells, which are then used for clustering through the Leiden algorithm. We followed the tutorial in the SpaceFlow GitHub repository (https://github.com/hongleir/SpaceFlow). The default parameters were used. The model was trained for 1000 epochs using a learning rate of 0.001, a regularization strength parameter λ of 0.01, and a learned embedding size of 50.

GraphST. GraphST also builds upon the DGI framework [5]. It uses the PCA-processed reconstructed gene expression for clustering with mclust. GraphST employs a heuristic refinement step to rectify misassignments of spots to spatially disparate domains. We followed the documentation of GraphST (https://deepst-tutorials.readthedocs.io/en/latest/). The recommended parameters were used, including a learning rate of 0.001 and training epoch of 600. Here, we used the top 20 PCs for clustering. The cluster refinement method of GraphST was used for the spot-level data.

STAGUE. We used the Adam optimizer [7] with a learning rate from $\{0.0005, 0.001\}$ and a consistent weight decay of 0.001 across all datasets. For F_g larger than 1000, we set F to 512; otherwise, it was set to 256. However, for osmFISH with low gene coverage, F was set to 64. The output dimensions for the two layers in $f_o(\cdot)$ were set to F, whereas for $f_l(\cdot)$, they were defaulted to 256 and 64, respectively. The projection header's output dimension was set to a default

of 64. In constructing the raw adjacency matrix Γ, we set k_Γ to 5 for single-cell level data and 6 for spot-level data, reflecting the latter's hexagonal grid structure. The spatial decay rate hyperparameter γ was selected from $\{1, 2, 3\}$. The cosine similarity weight ω_o was set to 0.5. We used a default k of 15 for single-cell level data and 20 for spot-level data to determine the cutoff distance d_c. The adjustment rate ω_Γ for the raw adjacency matrix was fixed at 0.999. The feature masking rates $p^{(\cdot,r)}$ were selected from [0.0, 0.9], and the edge dropping rates $p^{(\cdot,e)}$ were set to an empirical value of 0.5. For the temperature hyperparameter τ, we set it to 0.3 for single-cell level ST data and 0.2 for spot-level ST data. The triplet loss margin ϵ was generally selected from [0.0, 1.5], and its weight ω_t from [0.5, 5.0]. We performed clustering using the learned embedding with the same procedure for DeepLinc. Here, we also applied GraphST's heuristic refinement step for spot-level data.

Notably, the comparison methods have utilized a variety of unsupervised clustering algorithms, such as k-means, mclust, Louvain, and Leiden, to perform clustering on the generated cell embeddings. In the benchmarking analysis, we observed that certain clustering algorithms demonstrate particular appropriateness for specific datasets. For instance, the k-means algorithm achieves higher scores with the mouse V1/mPFC dataset, while the mclust method excels when applied to the DLPFC dataset. Here, the evaluation procedure for DeepLinc was applied to STAGUE and other DL-based baselines as a standardized benchmarking workflow. Specifically, the classic k-means algorithm was employed to benchmark the cell embeddings, with each model being executed five times to determine the average performance. This approach ensures a fair comparison of the learned embeddings' representational capability and mitigate the variability introduced by the choice of clustering algorithms.

2 Supplementary Tables

Table S1: Summary of the Real#1 dataset group. Datasets from 10x Visium are spot-level, and the rest are single-cell resolution.

Platform	Tissue	Identifier	#Cells/Spots	#Genes	#Domains
STARmap	Mouse primary visual cortex	V1	1,207	1,020	7
	Mouse medial prefrontal cortex	BZ5	1,049	166	4
		BZ9	1,053	166	4
		BZ14	1,088	166	4
MERFISH	Mouse hypothalamic preoptic area	Bregma-0.04	5,488	155	8
		Bregma-0.09	5,557	155	8
osmFISH	Mouse somatosensory cortex	osmFISH	4,839	33	11
Stereo-seq	Mouse olfactory bulb	Stereo-seq	10,000	26,145	8
10x Visium	Human dorsolateral prefrontal cortex	151507	4,221	33,538	7
		151508	4,381	33,538	7
		151509	4,788	33,538	7
		151510	4,595	33,538	7
		151671	4,093	33,538	5
		151672	3,888	33,538	5

Table S2: Summary of the Real#2 dataset group. The dataset identifier corresponds to the one in http://sdmbench.drai.cn/.

Platform	Tissue	Identifier	#Spots	#Genes	#Domains
Spatial Transcriptomics	HER2-positive breast tumor	A1	341	15,045	5
		B1	269	15,109	4
		C1	167	15,557	3
		D1	255	15,661	3
		E1	534	15,701	3
		F1	659	14,861	3
		H1	530	15,029	6
10x Visium	Liver	JBO1	1,293	31,053	4
		JBO2	1,363	31,053	4
		JBO3	1,316	31,053	4
		JBO4	1,790	31,053	4
		JBO6	1,121	31,053	4
		JBO9	2,002	31,053	4
		JBO10	1,780	31,053	4
Spatial Transcriptomics	Pancreatic ductal adenocarcinoma	GSM3036911	428	19,738	5
		GSM3405534	224	19,738	5

Table S3: Simulation configurations for the Simulated#2 dataset group. The name of the simulation aligns with that mentioned in scSpace [8].

Simulation	#Clusters	#Subclusters	#Total Cell Types	#Cells
sim1	2	2	3	1,000
sim2	2	3	4	1,000
sim3	2	4	5	1,000
sim4	3	2	4	1,000
sim5	3	3	5	1,000
sim6	3	4	6	1,000
sim7	4	2	5	1,000
sim8	4	3	6	1,000
sim9	5	3	7	1,000
sim10	5	4	8	1,000
sim51	2	5	6	1,000
sim52	3	5	7	1,000
sim53	5	5	9	1,000
sim66	2	6	7	1,000
sim67	3	6	8	1,000
sim68	5	6	10	1,000
sim81	2	7	8	1,000
sim82	3	7	9	1,000
sim83	5	7	11	1,000
sim96	2	8	9	1,000
sim97	3	8	10	1,000
sim98	5	8	12	1,000
sim111	2	9	10	1,000
sim112	3	9	11	1,000
sim113	5	9	13	1,000
sim126	2	10	11	1,000
sim127	3	10	12	1,000
sim128	5	10	14	1,000

3 Supplementary Figures

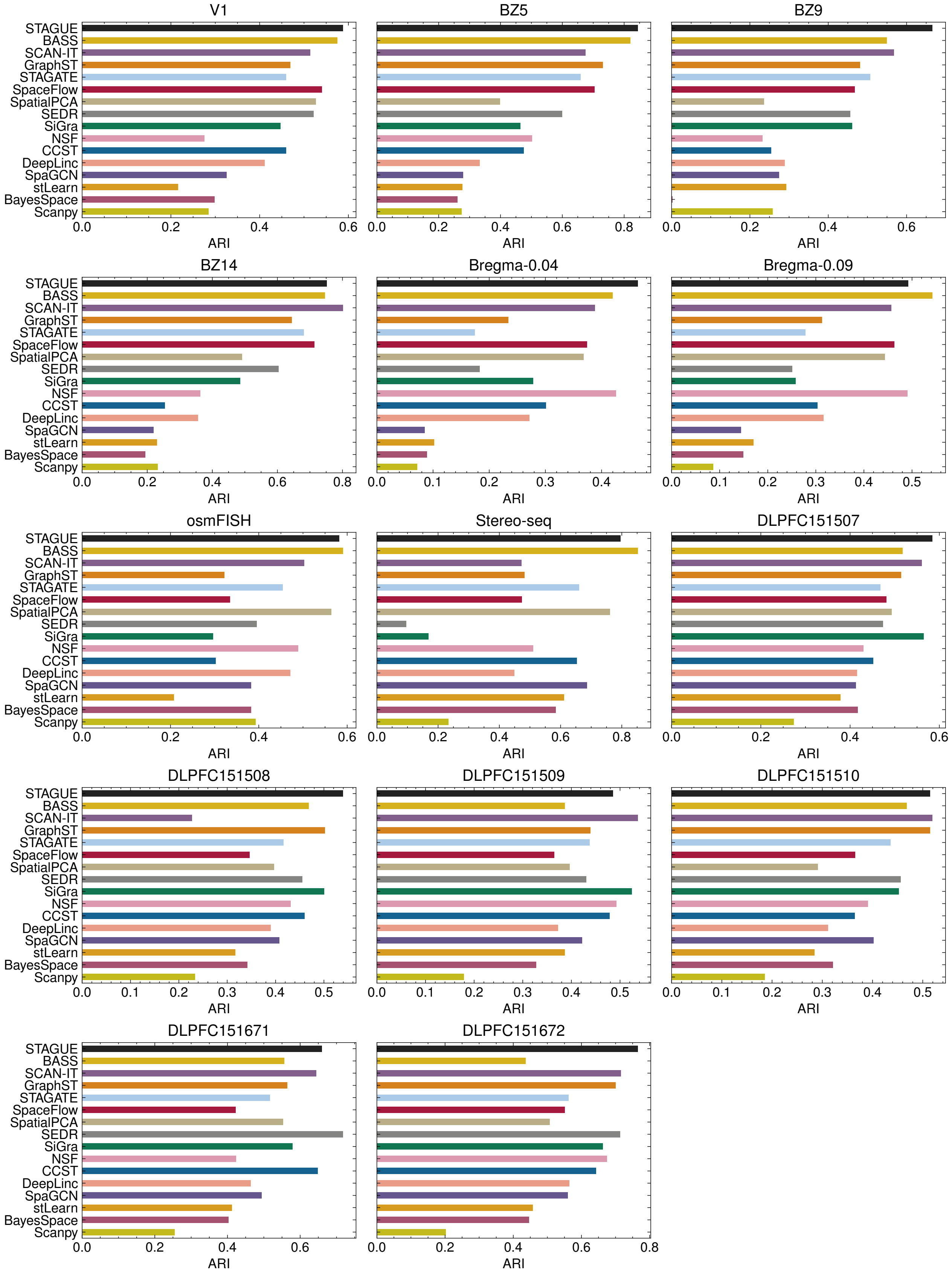

Figure S1: ARI performance comparison of all methods on the Real#1 dataset. We run all the deep learning methods five times and provide the mean values.

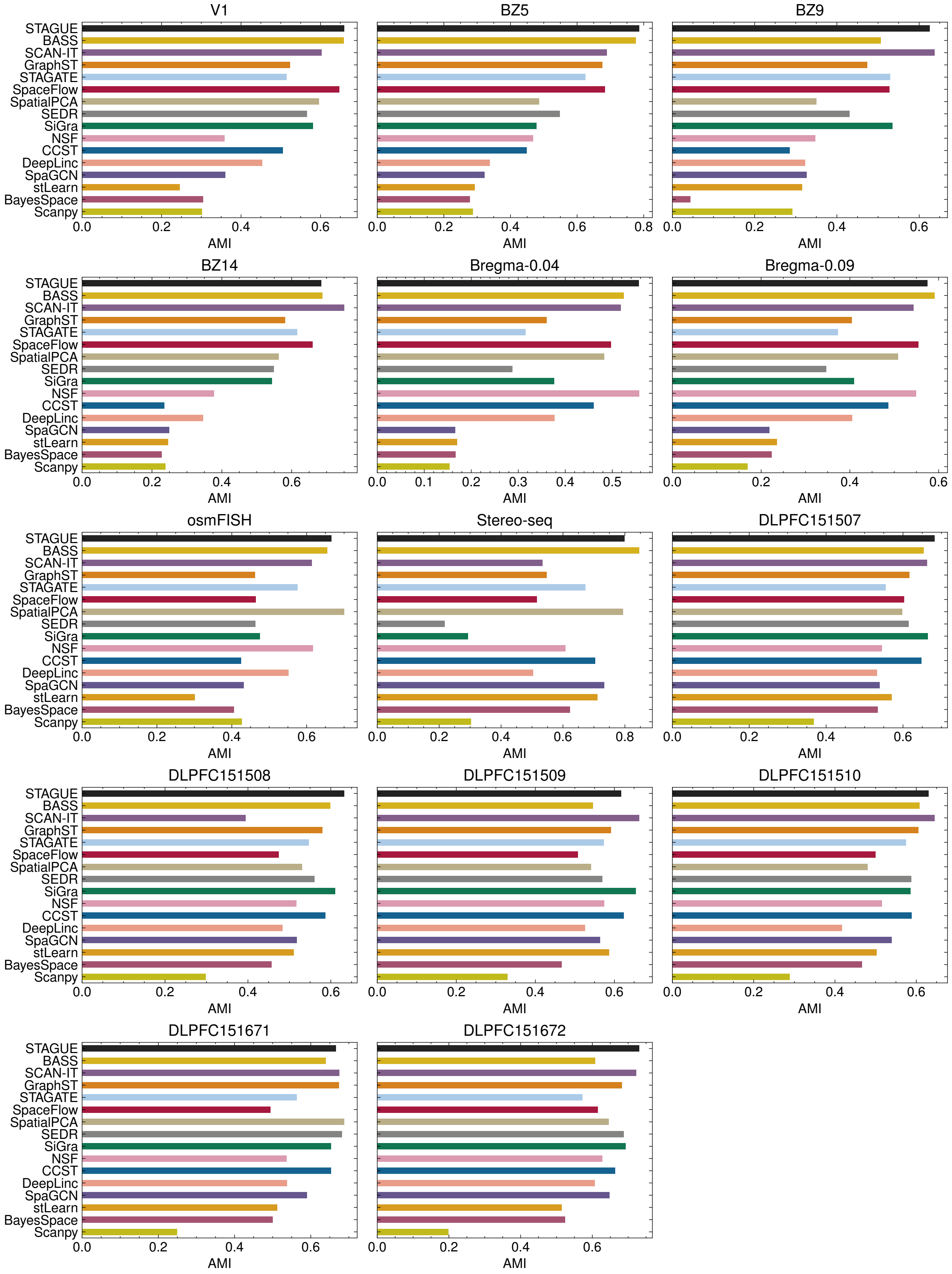

Figure S2: AMI performance comparison of all methods on the Real#1 dataset. We run all the deep learning methods five times and provide the mean values.

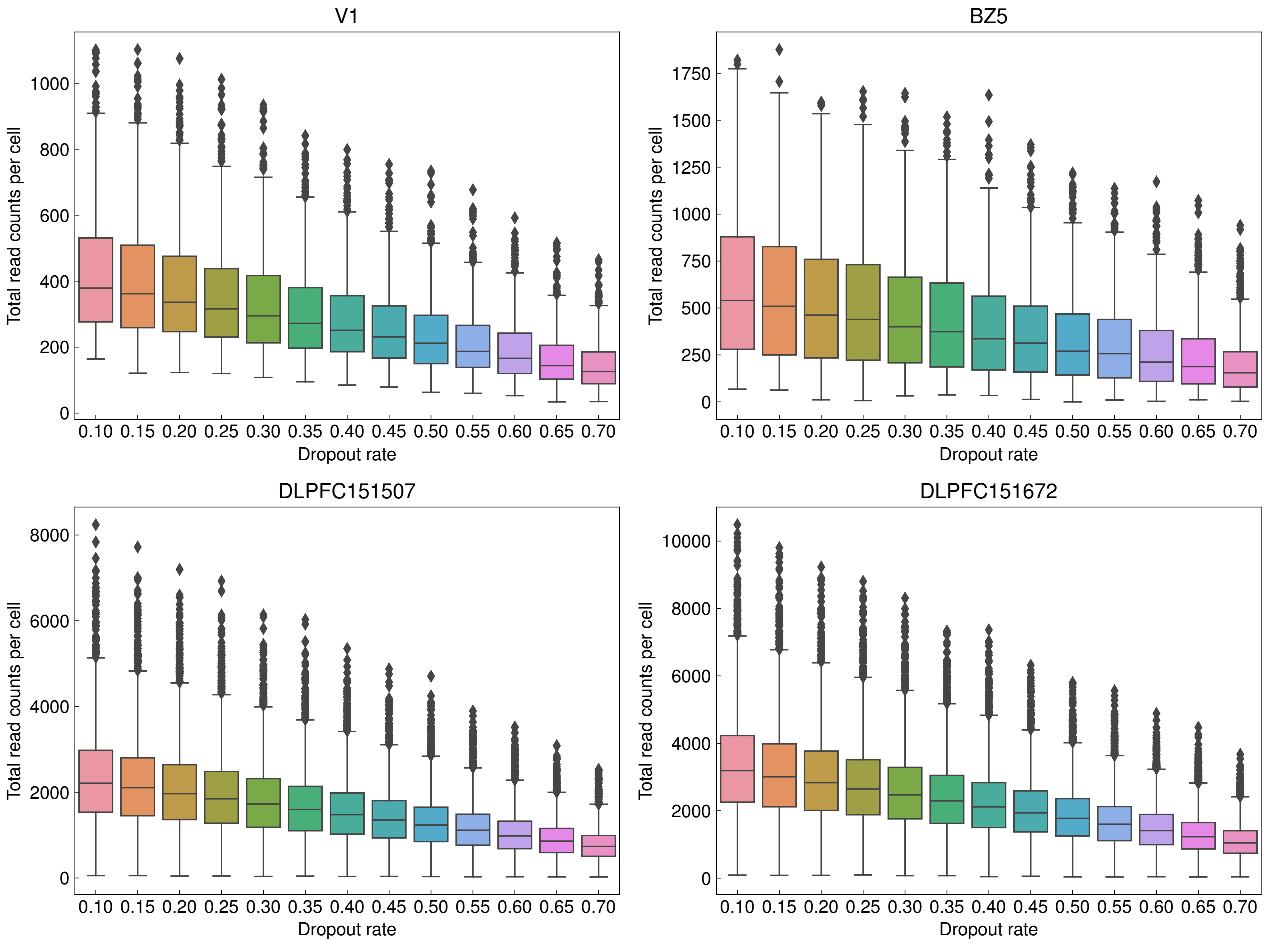

Figure S3: Sequencing depths after downsampling with an increasing dropout rate from 0.1 to 0.7.

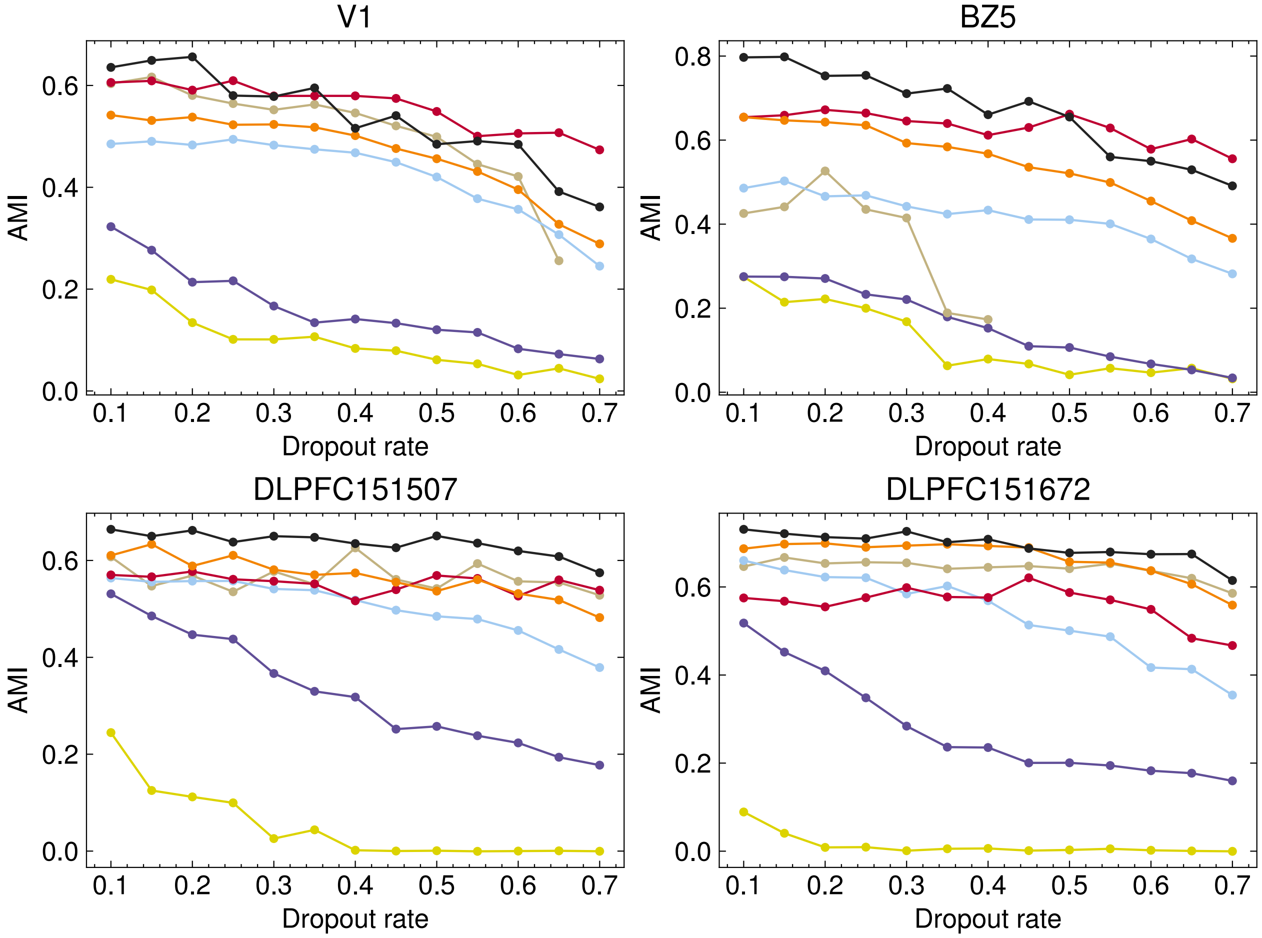

Figure S4: AMI performance of selected representative methods under different dropout rates, ranging from 0.1 to 0.7 with a step of 0.05.

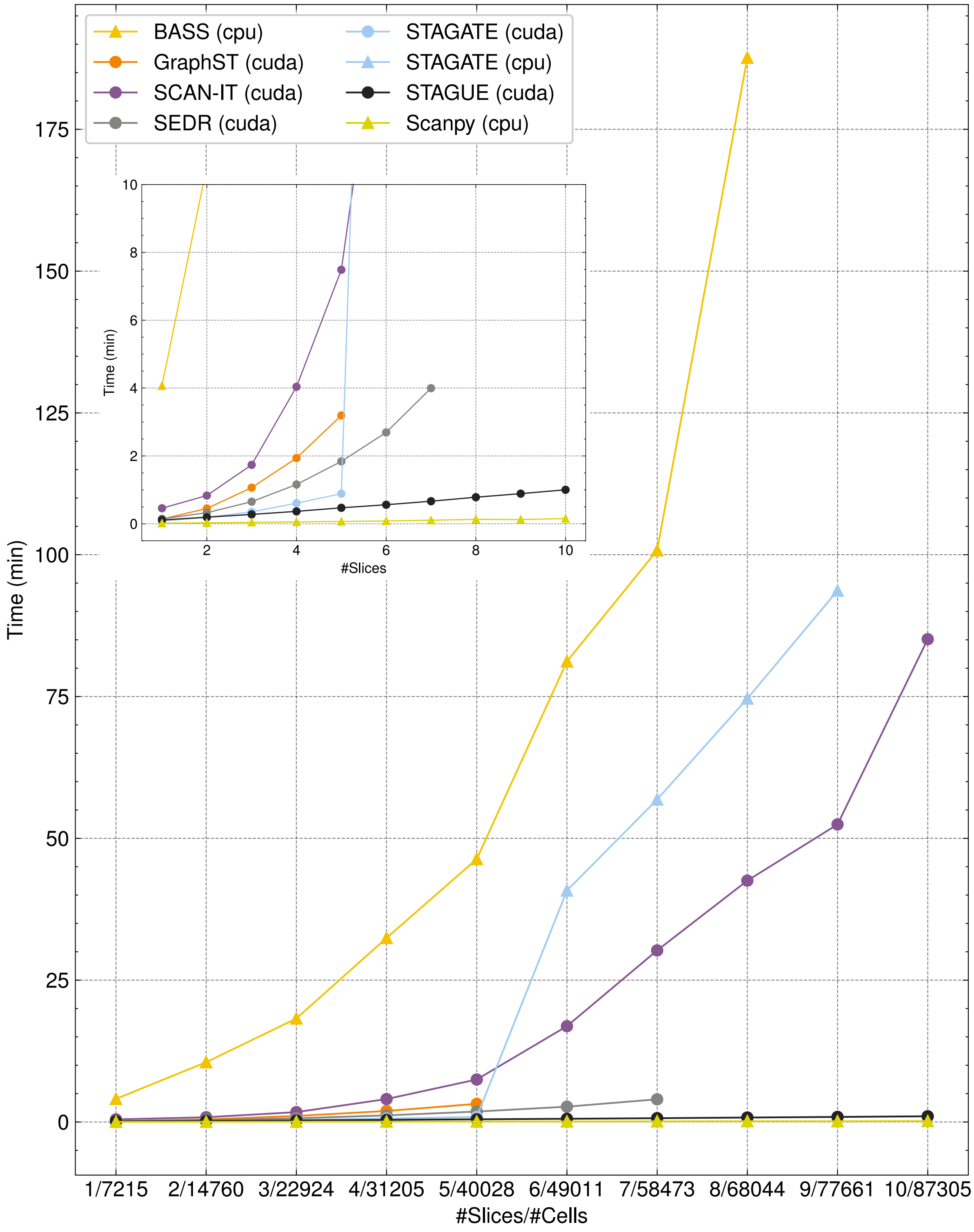

Figure S5: Scalability analysis of representative methods. The subfigure details the running times of less than 10 minutes. Not all methods are applicable across the 10 slices, as some encounter out-of-CUDA-memory errors. STAGATE can continue running on the CPU.

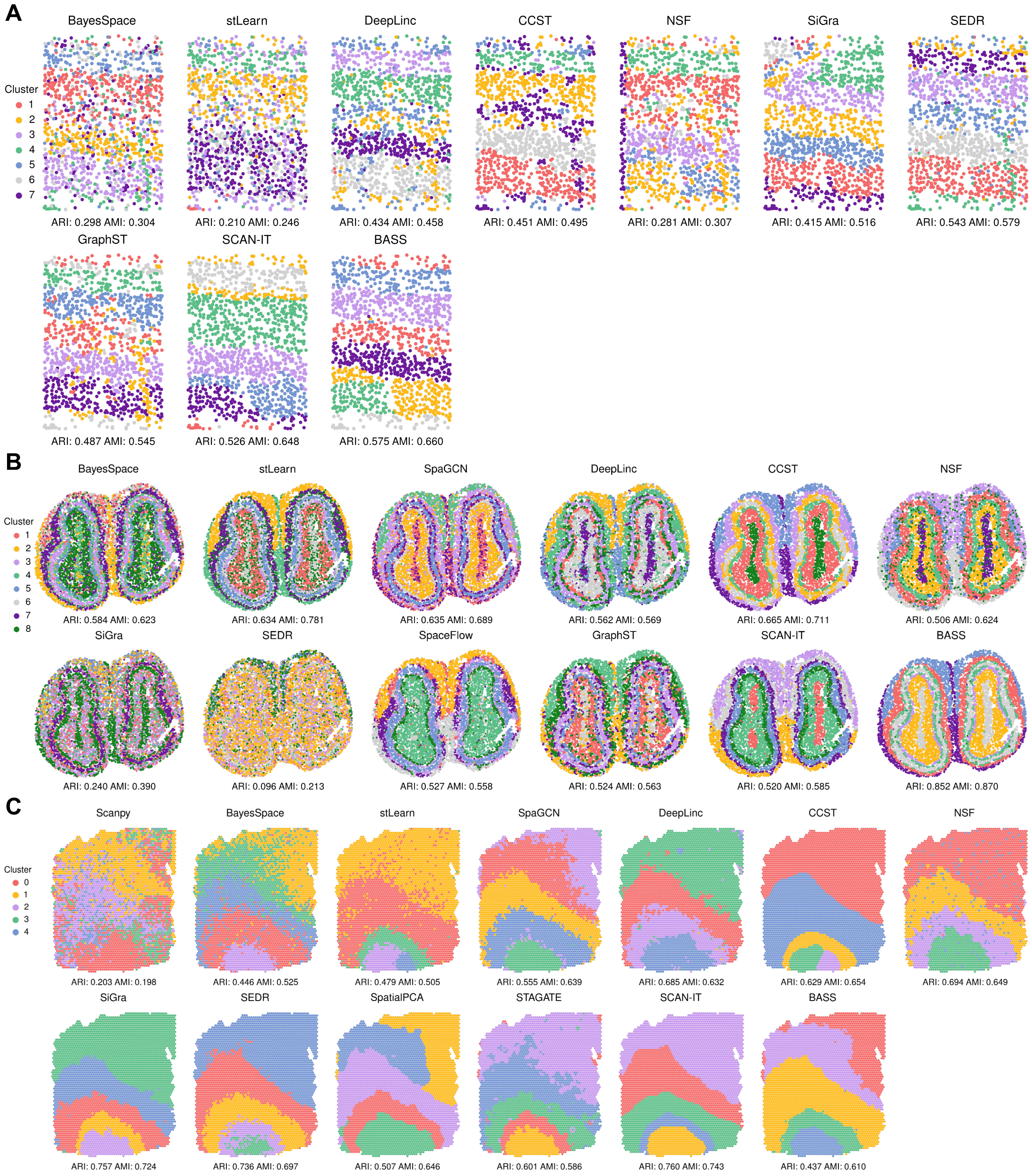

Figure S6: Clustering results of different methods for datasets **(A)** V1, **(B)** Stereo-seq, and **(C)** slice #151672 of DLPFC. Performance from a single run of each method is selected for demonstration.

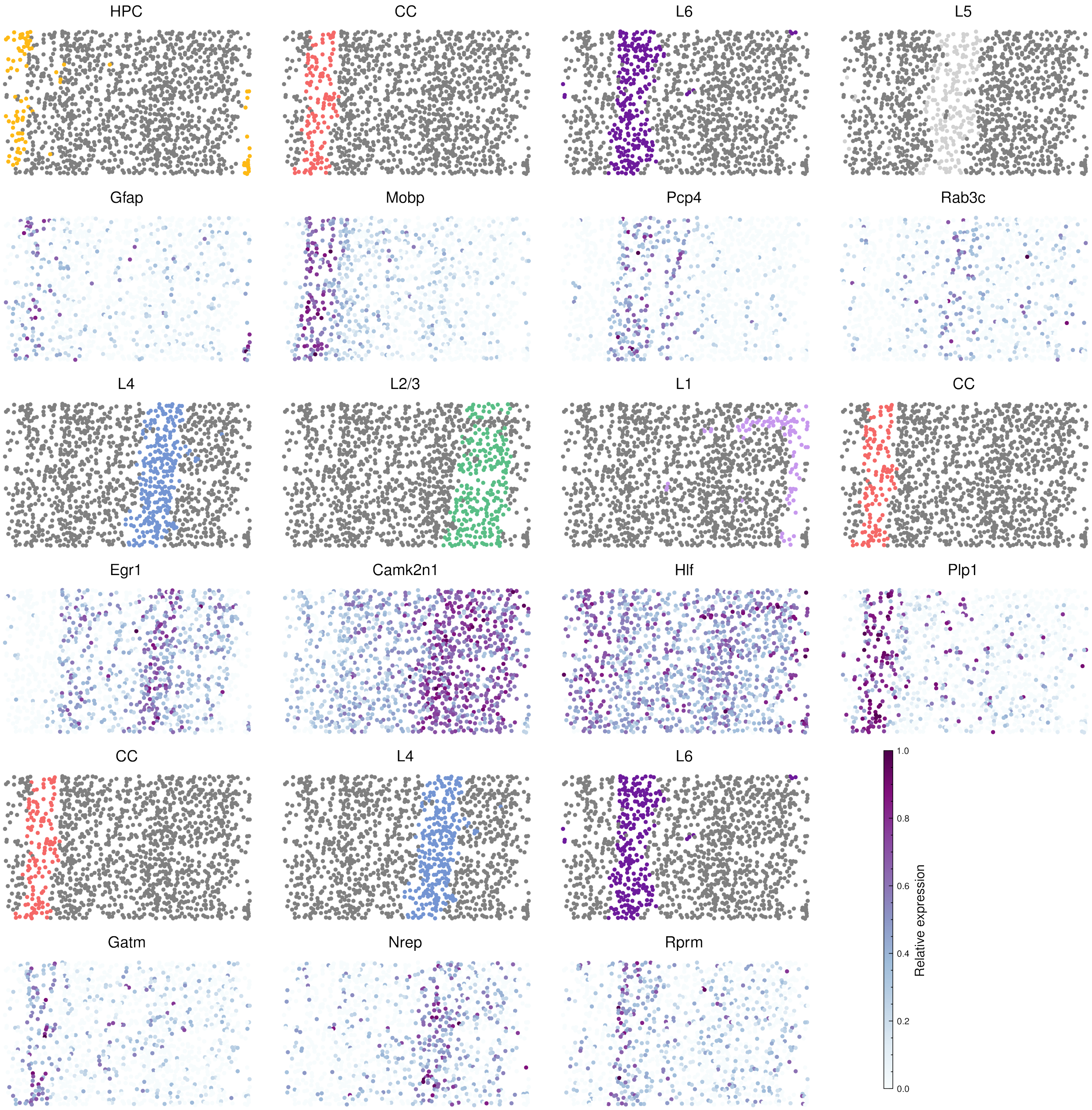

Figure S7: Expression patterns of differentially expressed genes of different clusters identified by STAGUE in the V1 dataset.

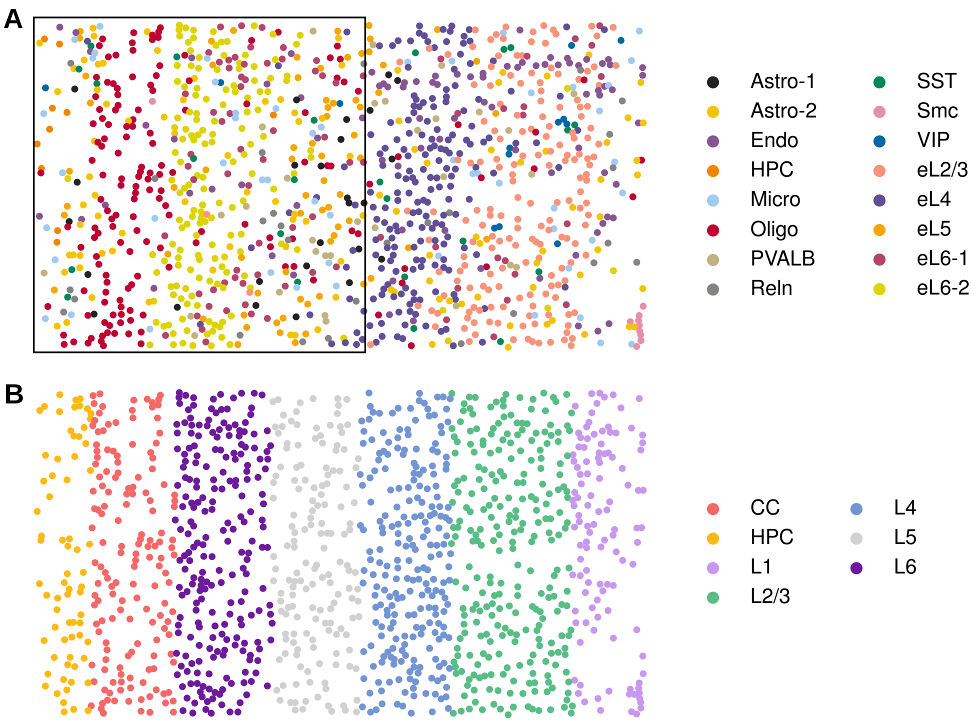

Figure S8: **(A)** Cell type distribution and **(B)** manual annotations of the domains for the STARmap V1 dataset. The oligodendrocytes demonstrate great dispersion across domains within the black box. Astro, astrocytes; Endo, endothelial cells; HPC, hippocampal excitatory subtype; Micro, microglia; Oligo, oligodendrocytes; PVALB, Reln, SST, VIP, inhibitory neuron subtypes; Smc, smooth muscle cells; eL2/3, eL4, eL5, eL6, excitatory neuron subtypes.

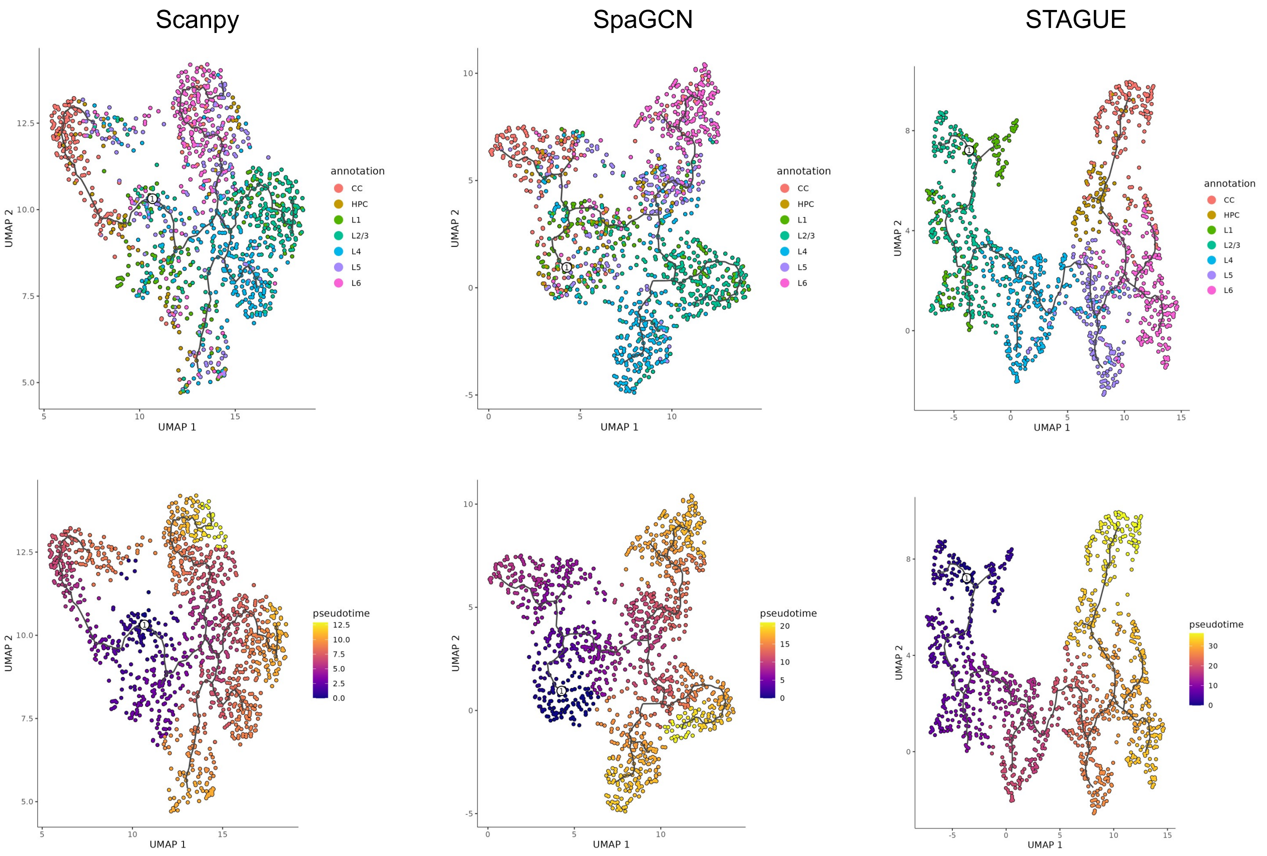

Figure S9: Comparison of pseudo-time trajectories inferred using Monocle3 based on latent embeddings from Scanpy, SpaGCN, and STAGUE. L1 was designated as the root. UMAP plots colored by ground-truth labels (top) and pseudo-time (bottom), with trajectories overlaid.

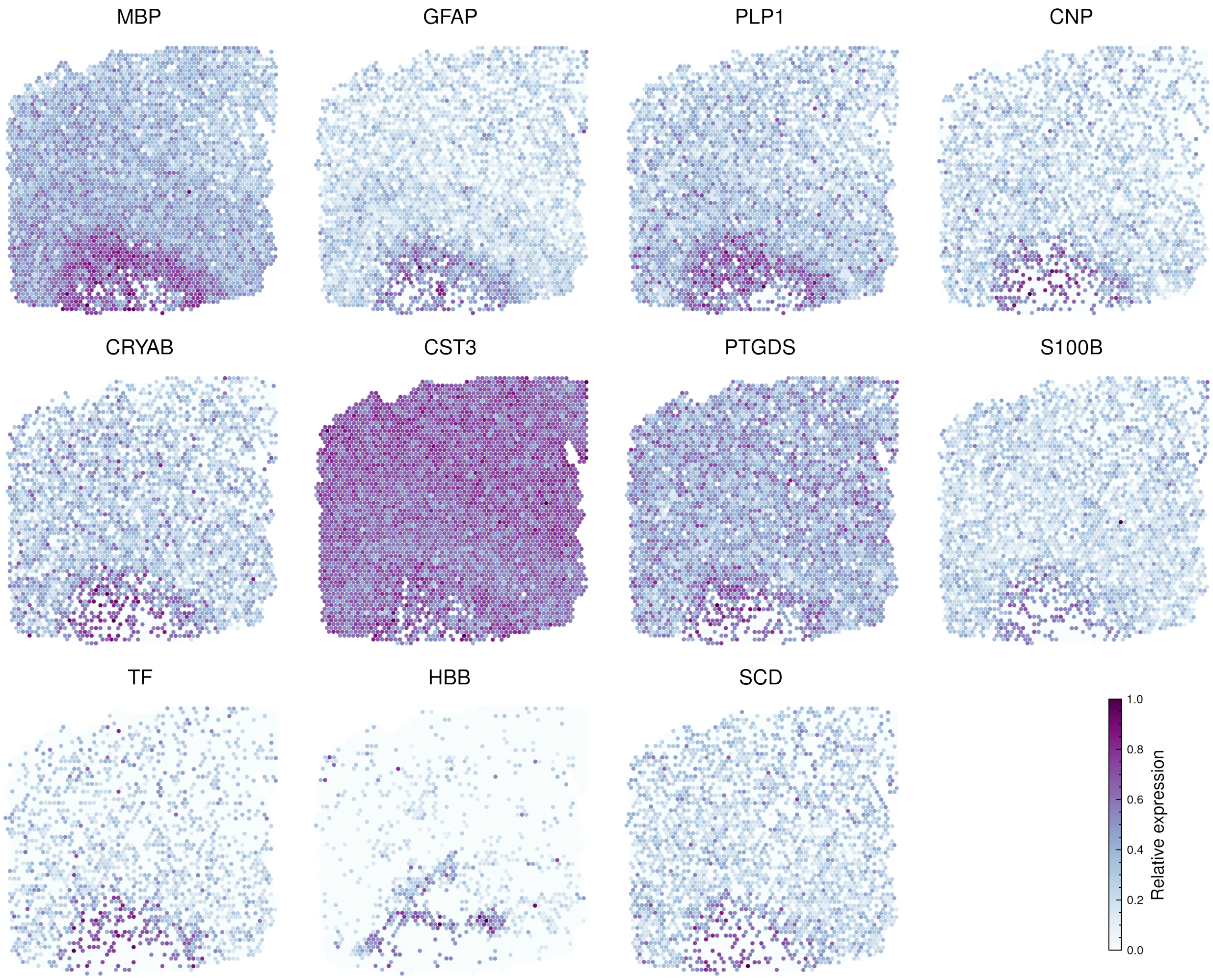

Figure S10: Expression patterns of the representative differentially expressed genes between clusters 0 and 3/4 identified by STAGUE in slice #151672 of the DLPFC dataset.

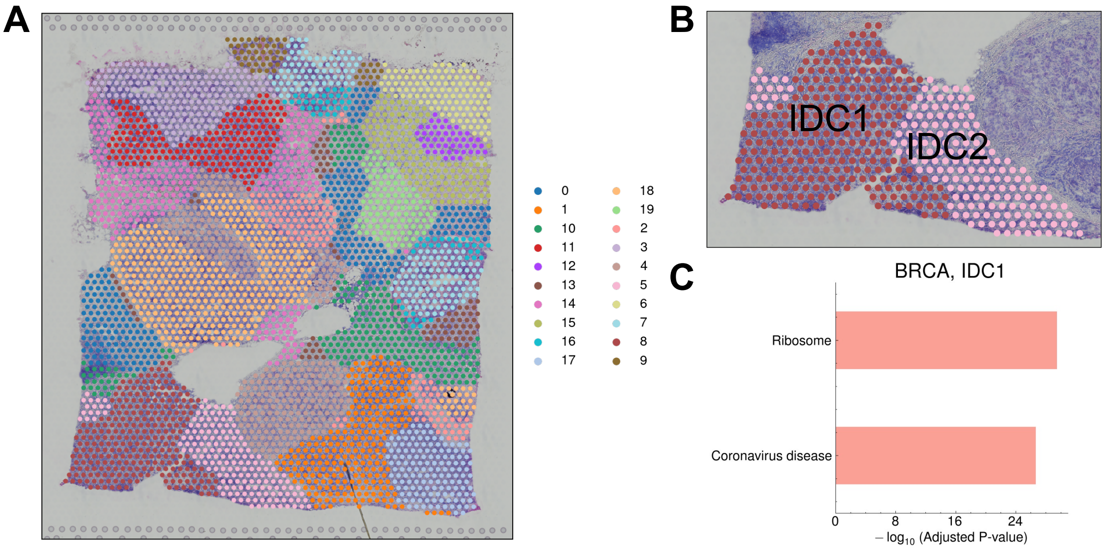

Figure S11: **(A)** Clustering result of STAGUE with $n_c = 20$. **(B)** STAGUE identified two sub-regions within the IDC region located at the bottom left corner. **(C)** Pathway enrichment analysis of the IDC1 sub-region.

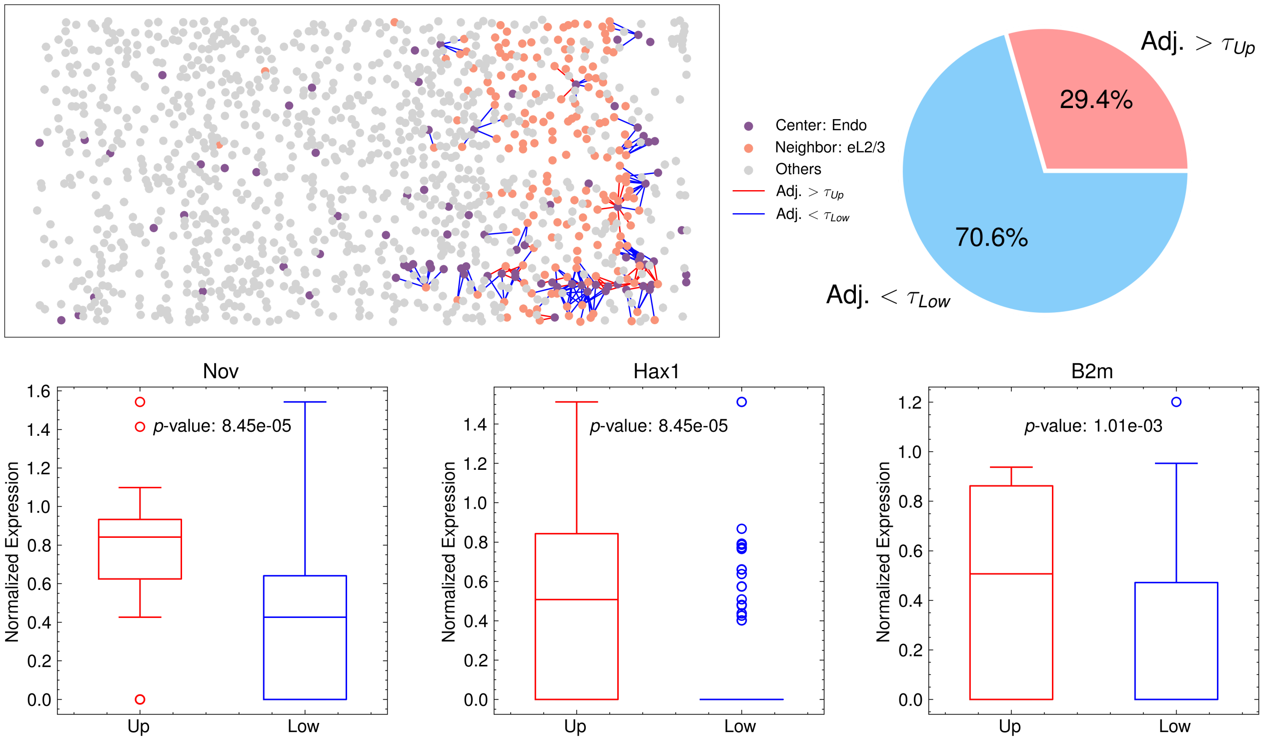

Figure S12: Comparison of stronger and weaker CCIs between endothelial cells and eL2/3 cells, including interaction ratios and top upregulated genes in eL2/3 cells with stronger CCIs.

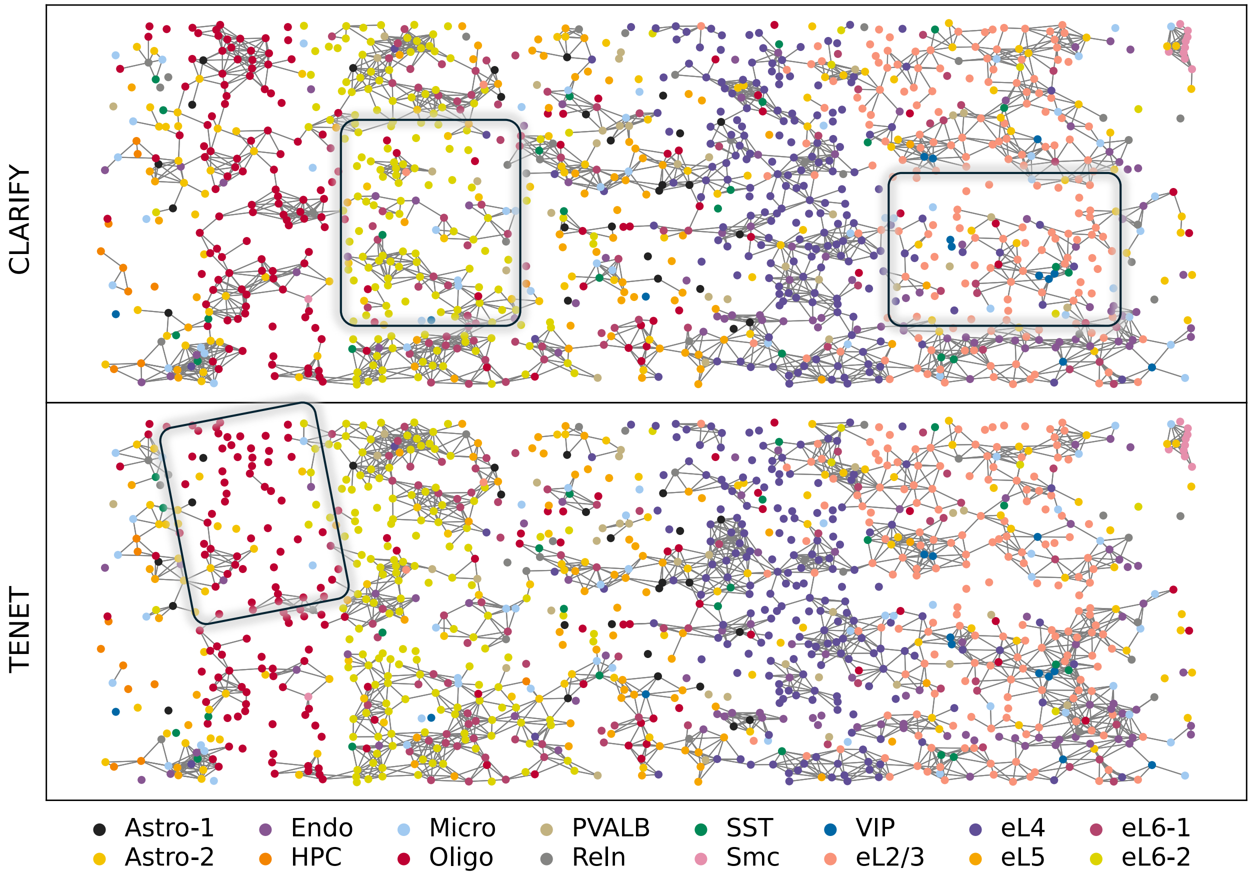

Figure S13: CCI inference results on the STARmap V1 dataset using CLARIFY and TENET, with edge counts of 5,382 and 5,376. Astro, astrocytes; Endo, endothelial cells; HPC, hippocampal excitatory subtype; Micro, microglia; Oligo, oligodendrocytes; PVALB, Reln, SST, VIP, inhibitory neuron subtypes; Smc, smooth muscle cells; eL2/3, eL4, eL5, eL6, excitatory neuron subtypes.

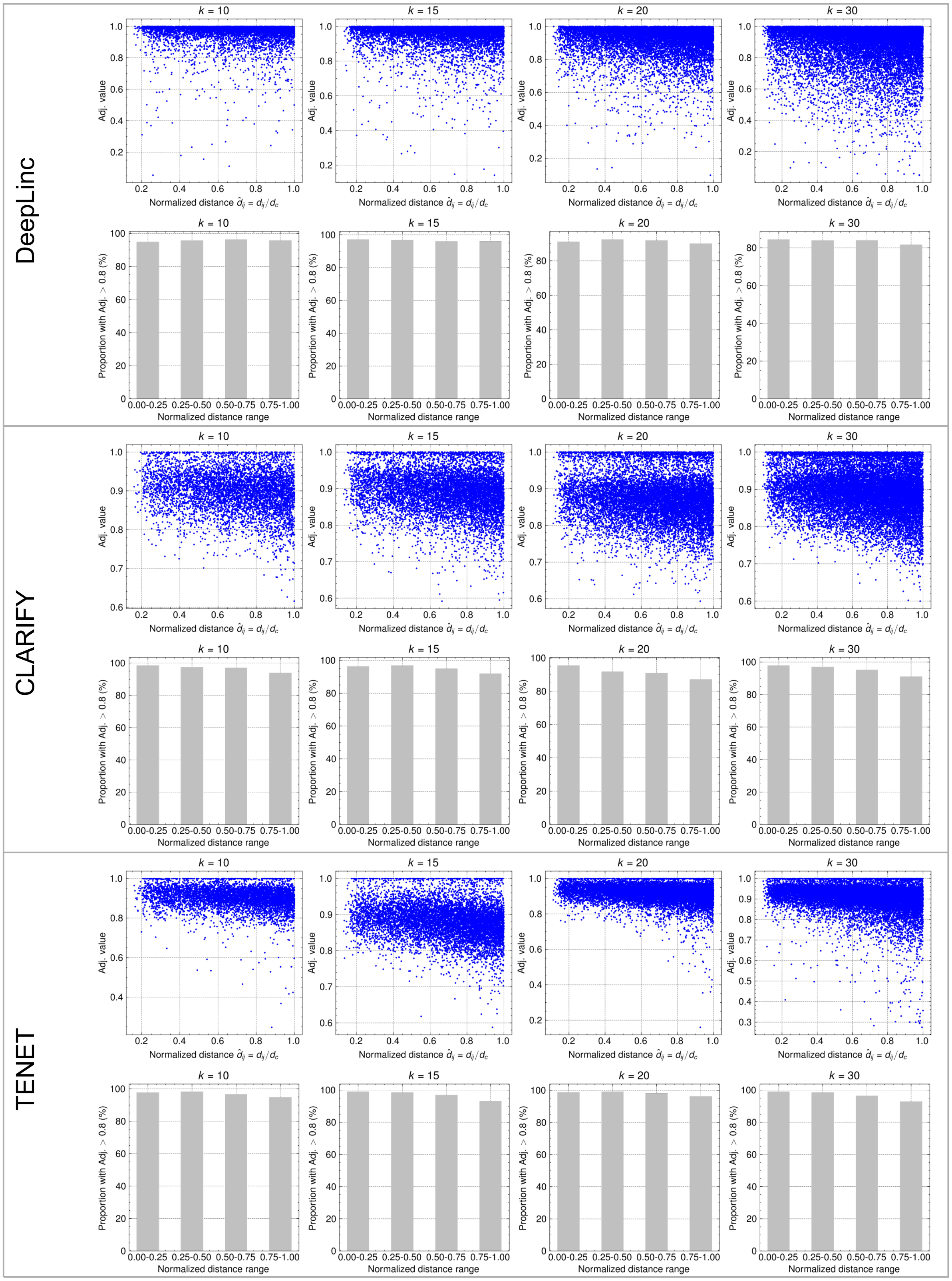

Figure S14: Values in the learned adjacency matrices from DeepLinc, CLARIFY, and TENET (top). Proportion of adjacency values exceeding 0.8 across various ranges of normalized distance (bottom). The threshold of 0.8 was chosen because their adjacency values are predominantly distributed at high levels.

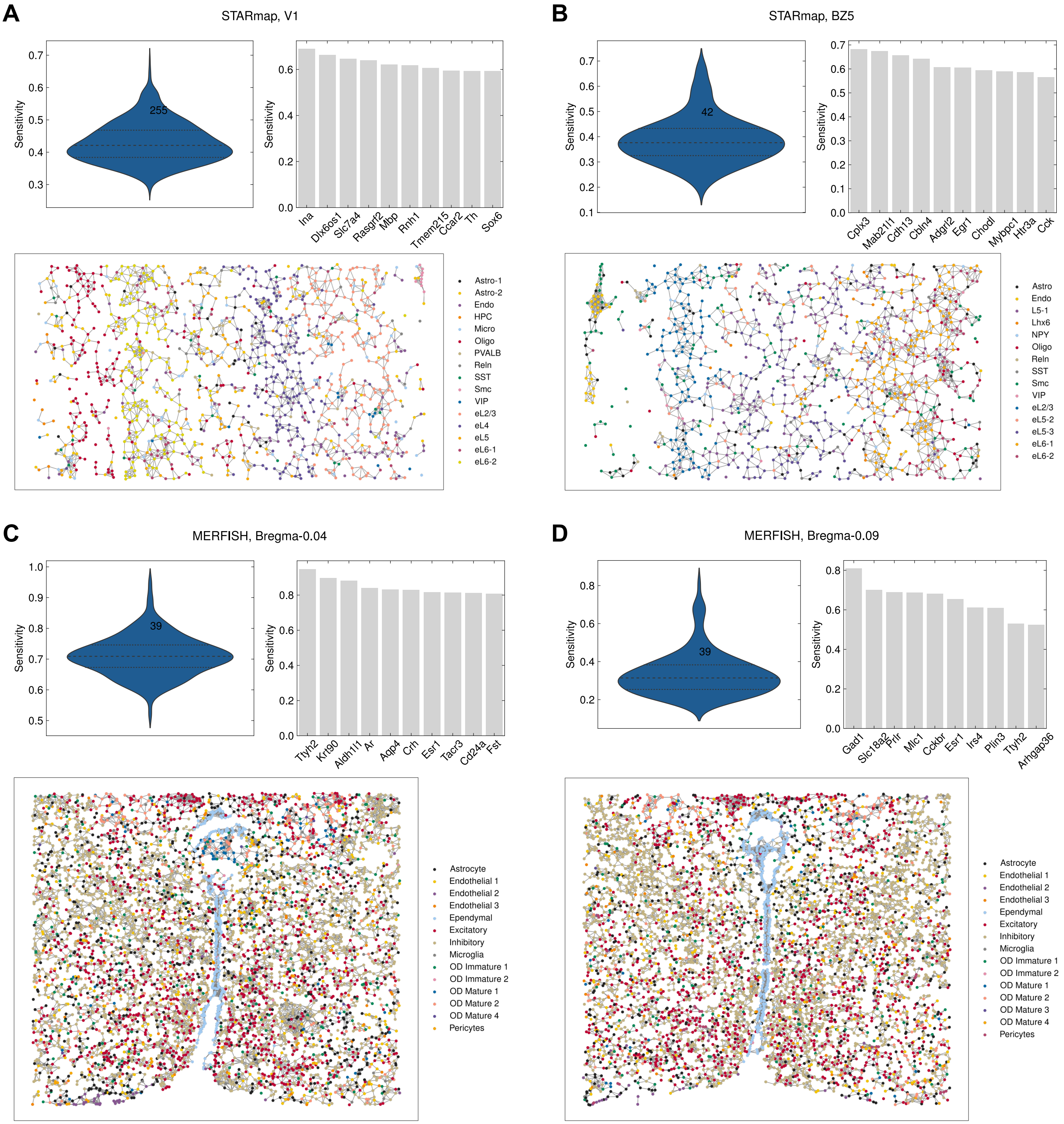

Figure S15: Distribution of sensitivity scores (violin plot), the top 10 sensitive genes (bar plot), and the learned cell graph adjacency matrix from the original expression data of four datasets: **(A)** V1, **(B)** BZ5, **(C)** Bregma-0.04, and **(D)** Bregma-0.09. Genes that have sensitivity scores in the top 25% were designated as signature genes, with their counts displayed in the violin plots. A threshold of 0.65 was used to establish the edges in the cell graph adjacency matrix.

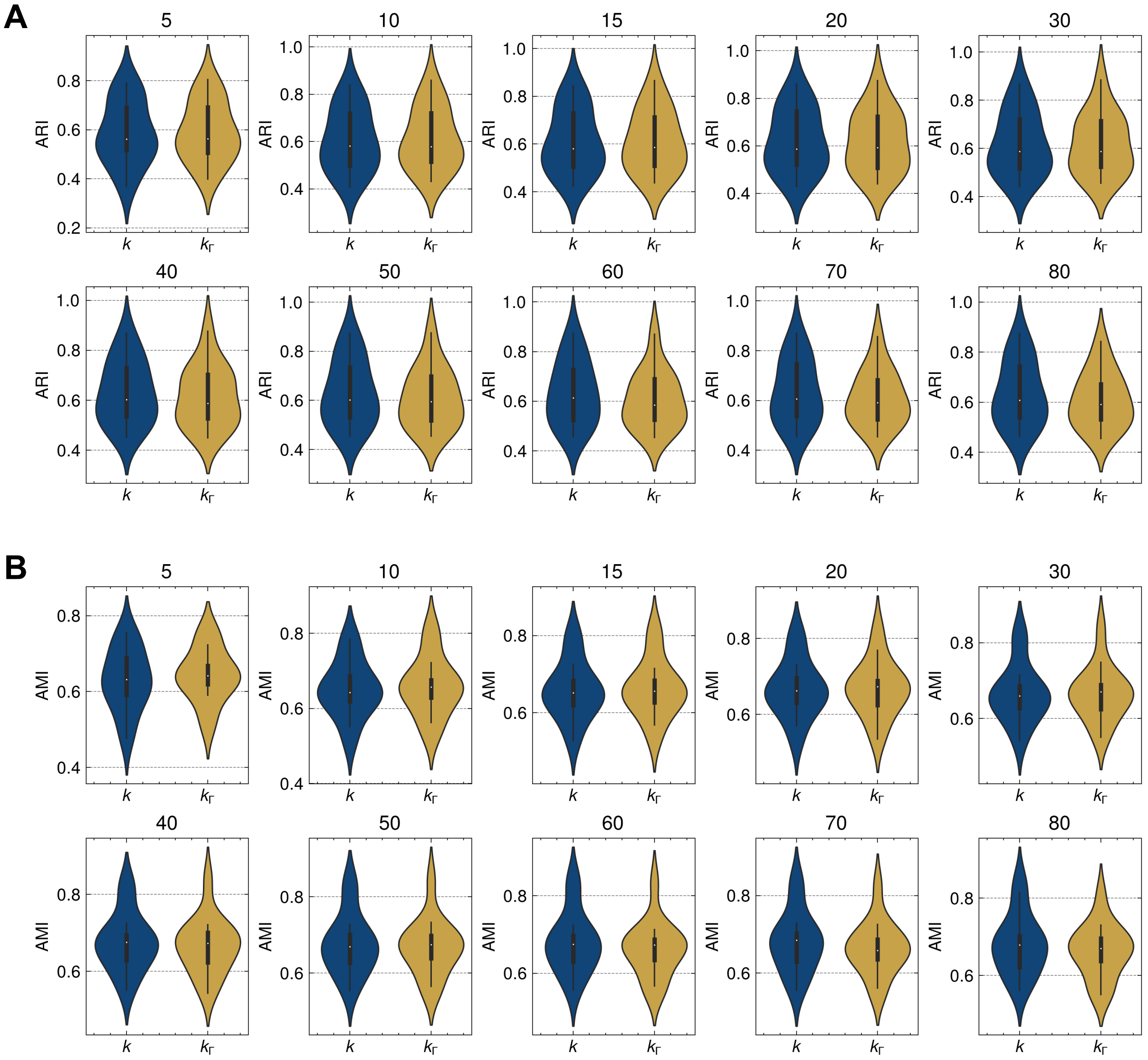

Figure S16: **(A)** ARI and **(B)** AMI distribution corresponding to each value of k_Γ and k.

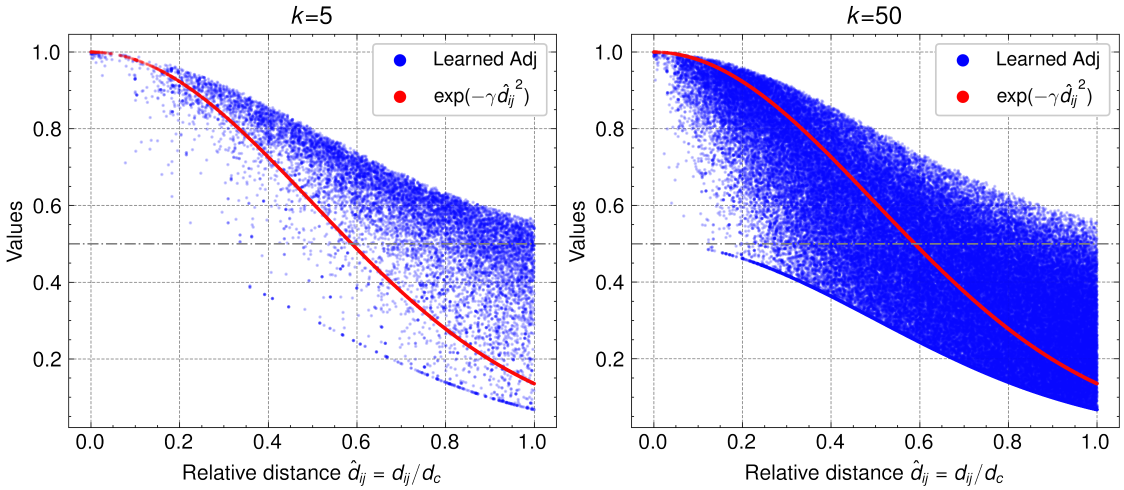

Figure S17: Values of the learned adjacency (before normalization) and the spatial decay term in the osmFISH dataset. The gray dashed line represents the 0.5 threshold. The parameter d_c corresponds to the median distance between each cell and its k-th nearest neighbor. Here, $\gamma = 2$.

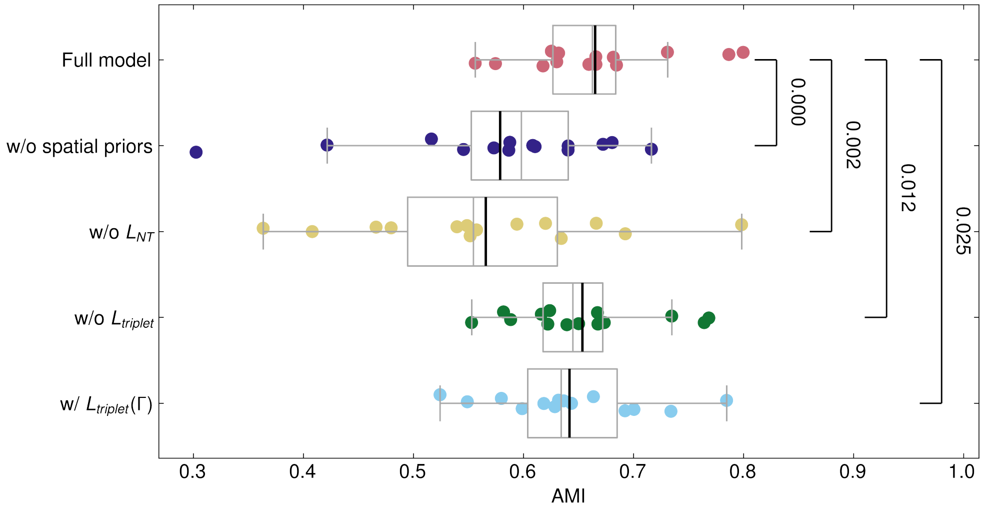

Figure S18: AMI performance of STAGUE with different settings. The p-value was calculated with the one-sided Wilcoxon signed-rank test. Each point represents the result of the corresponding method on one dataset. The black center line indicates the mean value across all datasets. Boxplot: center line, the median; upper and lower edges, the interquartile range; whiskers, 1.5 × interquartile range.

References

1. Traag, V. A., Waltman, L. & Van Eck, N. J. From Louvain to Leiden: guaranteeing well-connected communities. *Scientific reports* **9,** 5233 (2019).
2. Fraley, C., Raftery, A. E., Murphy, T. B. & Scrucca, L. *mclust version 4 for R: normal mixture modeling for model-based clustering, classification, and density estimation* tech. rep. (Technical report, 2012).
3. Xie, J., Girshick, R. & Farhadi, A. *Unsupervised deep embedding for clustering analysis* in *International conference on machine learning* (2016), 478–487.
4. Pan, S. *et al. Adversarially Regularized Graph Autoencoder for Graph Embedding* in *Proceedings of the 27th International Joint Conference on Artificial Intelligence* (2018), 2609–2615.
5. Veličković, P. *et al. Deep Graph Infomax* in *International Conference on Learning Representations* (2019).
6. Veličković, P. *et al. Graph Attention Networks* in *International Conference on Learning Representations* (2018).
7. Kingma, D. & Ba, J. *Adam: A Method for Stochastic Optimization* in *International Conference on Learning Representations* (2015).
8. Qian, J. *et al.* Reconstruction of the cell pseudo-space from single-cell RNA sequencing data with scSpace. *Nature Communications* **14,** 2484 (2023).
